# SPARC coordinates extracellular matrix remodeling and efficient recruitment to and migration of antigen-specific T cells in the brain following infection

**DOI:** 10.1038/s41598-021-83952-0

**Published:** 2021-02-25

**Authors:** Kathryn E. McGovern, J. Philip Nance, Clément N. David, Reed E. S. Harrison, Shahani Noor, Danielle Worth, Tyler A. Landrith, Andre Obenaus, Monica J. Carson, Dimitrios Morikis, Emma H. Wilson

**Affiliations:** 1 Division of Biomedical Sciences, School of Medicine, University of California, Riverside, Riverside, CA 92521 USA; 2 Department of Bioengineering, University of California, Riverside, Riverside, CA 92521-0129 USA; 3 School of Medicine, University of California, Irvine, Irvine, CA 92697 USA; 4 Present Address: BIO5 Institute, Department of Immunobiology, University of Arizona, Tucson, AZ 85724 USA; 5 Present Address: Nanostring Technologies, Inc, 530 Fairview Ave N, Seattle, WA 98109 USA; 6 Present Address: UCSD Bioengineering and the Institute for Engineering in Medicine, San Diego, CA 92093 USA; 7 Present Address: School of Medicine, MSC08, University of New Mexico, Albequerque, NM 87131 USA; 8 Present Address: Ambrey Genetics, Aliso Viejo, CA 92656 USA

**Keywords:** Parasite host response, Neuroimmunology

## Abstract

Central nervous system (CNS) injury and infection can result in profound tissue remodeling in the brain, the mechanism and purpose of which is poorly understood. Infection with the protozoan parasite *Toxoplasma gondii* causes chronic infection and inflammation in the brain parenchyma. Control of parasite replication requires the continuous presence of IFNγ-producing T cells to keep *T. gondii* in its slowly replicating cyst form. During infection, a network of extracellular matrix fibers, revealed using multiphoton microscopy, forms in the brain. The origin and composition of these structures are unknown but the fibers have been observed to act as a substrate for migrating T cells. In this study, we show a critical regulator of extracellular matrix (ECM) remodeling, Secreted Protein, Acidic, Rich in Cysteine (SPARC), is upregulated in the brain during the early phases of infection in the frontal cortex. In the absence of SPARC, a reduced and disordered fibrous network, increased parasite burden, and reduced antigen-specific T cell entry into the brain points to a role for SPARC in T cell recruitment to and migration within the brain. We also report SPARC can directly bind to CCR7 ligands CCL19 and CCL21 but not CXCL10, and enhance migration toward a chemokine gradient. Measurement of T cell behavior points to tissue remodeling being important for access of immune cells to the brain and facilitating cellular locomotion. Together, these data identify SPARC as an important regulatory component of immune cell trafficking and access to the inflamed CNS.

## Introduction

A functional immune response critically depends on the ability of effector cells to migrate to sites of infection. This process is tightly regulated and involves activation of innate and adaptive immune cells, production of cytokines and chemokines, and migration of these cells from the bloodstream into tissues^1,2^. Once within the tissue the mechanisms of cell migration remain poorly defined but can be dependent on cell type, tissue type, and the microenvironment^3^. Using multiphoton microscopy to image within live tissue, experiments can be conducted to observe the migration of fluorescently-tagged cells through tissues in real time^4^. In addition, components of the extracellular matrix (ECM) can be revealed through a phenomenon known as second harmonic generation (SHG). SHG can be used to visualize non-centrosymmetric structures, the most common of which are collagen fibers^5^. Studies using this technique highlight the importance of tissue matrix for the migration of immune cells within the lymph node, skin and tumors^6–11^. Indeed, the composition and density of collagen instructs interstitial migration at the cellular and molecular level with loosely packed collagen facilitating non-proteolytic and integrin independent migration^12^. These studies and others have allowed our understanding of the ECM to evolve from a static structural scaffold to a dynamic and receptive network capable of modulating cell migration, cell signaling, as well as the bioavailability of effector molecules^13^.

The structure and function of the ECM is regulated by matricellular proteins that modulate its assembly, degradation, maintenance, and physical properties^14^. These proteins, distinguished by their ability to regulate cell–matrix interactions, include thrombospondins, matrix metalloproteases (MMP), osteopontin, and the glycoprotein SPARC (secreted protein acidic and rich in cysteine)^15^. SPARC is a multi-functional molecule best known for its ability to bind many types of collagen in response to tissue damage or disease as well as regulate collagen deposition during development^16,17^. SPARC optimizes collagen assembly and crosslinking by binding collagen in a sequence specific manner^18^ and is thought to act as a chaperone for assembly of collagen during tissue remodeling^19,20^. In addition, SPARC can bind and interact with integrins, growth factors and cytokines. SPARC’s ability to inhibit VCAM-1 interactions facilitates breaks in endothelial cell barriers^21,22^ therefore may be a critical component of the inflammatory process. As well as changes in the ECM, SPARC may have direct effects on immune cells including encouraging NET formation by neutrophils and polarization of macrophages to M1^23,24^. Thus SPARC affects a multitude of cellular functions^25^, particularly cell migration as studied in models of cancer, inflammation, and infection. Indeed the more SPARC present in a tumor the more the tumor is prone to metastasis highlighting the clinical role of this molecule^26–29^.

Infection with the intracellular protozoan parasite *T. gondii* requires a continuous immune response in the brain for the lifetime of the host to prevent parasite reactivation and fatal pathology^30–33^. The ability of IFNγ-producing T cells to migrate to sites of infection in the brain is paramount in controlling the replication of the parasite. Work by this lab and others have shown chemokines like CCL21, CCL19, and CXCL10 are upregulated in the brain^34–37^. These chemokines are thought to control distinct aspects of cell behavior such as crossing into the parenchyma^36^ and mediating Levy walk migration patterns to efficiently encounter infected cells^34^. Additionally, multiphoton imaging and SHG of chronically infected brains revealed the presence of reticular fibers that are absent in the naïve brain^37^. Migrating CD8^+^ T cells are observed in association with these structures. The composition and source of this major tissue remodeling event is unknown, however, we hypothesized that this network is a mechanism for effector cells to migrate to sites of infection within the brain.

Here we show that SPARC is upregulated in the brain following infection with *T. gondii*. This upregulation is observed in the frontal cortex of the brain, an area associated with parasite infection and replication. The kinetics of SPARC induction correlate with the second harmonic signal and the formation of the fibrous network. In the absence of SPARC, mice have disrupted tissue remodeling, severely reduced infiltration of antigen-specific cells into the brain parenchyma and a failure to control parasite burden in the brain during early chronic infection. Furthermore, we find SPARC can directly bind T cell recruiting chemokines specifically CCR7 ligands. In chemotaxis assays, the presence of SPARC enhances CCL21-driven chemotaxis of lymphocytes. Moreover, intravital imaging reveals T cell access to the brain is patchy in the absence of SPARC and T cell migration is impaired. Taken together, these data point to an important role for SPARC in matrix remodeling in the early stages of CNS infection. In turn, this points to the blood brain barrier and tissue reorganization as being a major component of an appropriate, efficient, and protective immune response in the brain.

## Materials and methods

### Mice and parasites

The *T. gondii* Prugniaud strain expressing OVA, used to allow tracking of antigen specific T cells, was maintained in vitro as previously described, purified and mice were infected with 10,000 tachyzoites intraperitoneally^35^. The Me49 strain of *T. gondii* was maintained in infected Swiss Webster (SW-F, Taconic, Rensselaer, NY) and CBA/CaJ (000656, Jackson, Bar Harbor, ME) mice. For infection, brains from infected CBA/CaJ mice were removed, placed in 3 ml sterile 1xPBS and passed 3–5 times through an 18.5 gauge followed by 20.5 and 22.5 gauge needles. The number of cysts in a 30 μl aliquot was determined microscopically. Brain suspensions were adjusted to 100 cysts/ml and mice were infected each with 20 cysts intraperitoneally. C57Bl/6, CBA/CaJ and Swiss Webster mice were maintained in a specific pathogen free environment. All mice were housed and experimental procedures and methods were conducted in accordance with the ARRIVE guidelines and the Institutional Animal Care and Use Committee (IACUC). In addition, all methods and protocols were approved by the Biological Use Authorization Committee (BUA) at the University of California Riverside. SPARC-null (003728, Jackson, Bar Harbor, ME) mice were backcrossed with C57Bl/6 (000664, Jackson, Bar Harbor, ME) mice for at least 9 generations.

### Controlled cortical impact

To model inflammation during a traumatic brain injury (TBI), mice were anesthetized with isoflurane (3% induction, 1–2% maintenance) and placed in a stereotaxic frame to secure the head. Body temperature was maintained at 37 ± 1 °C with a heating pad during the surgery. A midline incision of the skin was made to expose the skull. A 5 mm craniotomy was carefully performed on the right side between Bregma and Lambda to expose the cortex. A moderate controlled cortical impact (CCI) was delivered using an electromagnetically driven piston (Leica Microsystems Company, Richmond, IL) with the following parameters: 1.5 mm depth, 3 mm diameter, 2.0 m/s speed, 200 ms dwell time. The skin was sutured and Buprenorphine (0.01 mg/kg, intramuscular) was administered after surgery to minimize pain. Sham animals underwent the whole procedure except for the impact. TBI animals were either sacrificed 1 or 7 days after the injury. Sham animals were sacrificed at 1 day.

### Preparation of splenocyte, lymph node, PECS and brain mononuclear cell (BMNC) suspensions

A single cell suspension from spleens and lymph nodes was prepared by passing through a nylon 40 μm cell strainer (BD, San Jose, CA). Suspensions were washed with RPMI complete (10% FCS, 1%Pennicilin/Streptomycin, 1% Glutamine, 1% Sodium Pyruvate, 1% nonessential amino acids, 0.1% B-mercaptoethanol) (Life Technologies, Grand Island, NY) and centrifuged for 5 min at 1200 RPM at 4 °C. Red blood cells were lysed using 0.86% ammonium chloride solution, centrifuged and resuspended in RPMI complete. Peritoneal exudate cells (PECS) were harvested by injecting 3 ml of sterile PBS into the peritoneal cavity, gently agitating the abdomen, and slowly drawing up the suspension with the syringe. BMNCs were prepared as previously described^38^. Prior to harvest, mice were intracardially perfused with 20 mL of ice-cold PBS, with perfusion confirmed by the white appearance of the brain and lack of red blood cells during tissue processing. Mononuclear cells were isolated from the brain by mincing and subsequent homogenization via passage through an 18-gauge needle in complete RPMI (86% RPMI, 10% FBS, 1% Pen/Strep, 1% L-glutamine, 1% NEAA, 1% Sodium pyruvate, < 0.01% β-mercaptoethanol). The resulting suspension was incubated at 37 °C with 3 mg DNAse and 100 μg collagenase for 1 h and 45 min. After incubation, the suspension was passed through a 70 μm strainer and mononuclear cells were isolated using a density gradient spun at 2000 rpm for 25 min with no brakes. The density gradient consisted of a 60% Percoll solution in cRPMI overlayed with a 30% Percoll solution in PBS. Brain mononuclear cells (BMNCs) were isolated from the interphase and counted using a haemocytometer.

### Flow cytometry

BMNC. PECs, lymph or splenic cells were washed in FACS buffer, blocked for 10 min in F_c_ block and incubated with various conjugated antibodies. To determine antigen specific T cells: CD3-FITC, OTI-CD8-dsRed, MHC I-Ova dextramer-APC (Immudex, Denmark and Tetramer core facility NIH, Bethesda, MD); to determine T cell activation: CD3-PerCp (145-2C11), CD4-PECy7(RM4-5), CD8-FITC (53-6.7), CD44-PE (IM-7), CD62L-APC (MEL-14); to determine monocyte populations: CD11c-APCCy7 (N418), CD11b-FITC (M1/70), Ly6C-PE (AL-21), Ly6G-APC (RB6-8C5), MHCII-PECy7 (M5/114.15.2), and CD45-PerCp (30F11), (eBioscience, San Diego, CA). Cells were analyzed using a BD FACSCanto II flowcytometer (BD Biosciences, San Jose, CA) and FlowJo analysis software v.8.7.3 (Treestar Software, Ashland, OR). Cell populations were determined by gating on CD4^+^, CD8^+^, (T cells) CD45^hi^/CD11b^+^ (macrophages) and CD45^int^/CD11b^+^ (microglia) Ly6CintLy6G^+^, (neutrophils) Ly6C^+^Ly6G^−^, (inflammatory monocytes) from a live cell gate (gating strategy as depicted in Supplementary Figs. 5, 7).

### Quantitative reverse transcription PCR (qRT-PCR)

To measure RNA from the cerebellum and cortex, brains were dissected into three basic parts from a sagittal cut half brain. The first cut was made from the base of the orbital lobe to the top of the cortex prior to lambda to achieve the cortical sample. The cerebellum was removed from the hindbrain to achieve the cerebellum sample. The rest of the brain was discarded. Total RNA from brain tissue samples was extracted with TRIzol reagent (Life Technologies, Grand Island, NY). DNase1 treatment and first strand cDNA synthesis was performed using cDNA synthesis kit (BioRad, Hercules, CA) according to the manufacturer’s instructions. SPARC-specific primers for Real Time PCR were purchased from IDT's primer Quest (http://www.idtdna.com/Scitools/ Applications/Primerquest/). Primer sequences were as follows: SPARC forward, 5′-ATTAGGCTGTTGGTTCAAA-3′; reverse, 5′-AGCCCTGGTTCTCCAAAA-3′; CCL21 forward, 5′-TGAGCTATGT GCAAACCCTGAGGA-3; reverse, 5′-TGAGGGCTGTGTCTGTTCAGT TCT-3′, CCL19 forward, 5′-ATGTGAATCACTCTGGCCCAGGAA-3′; reverse, 5′-AAGCGGCTTTATTGGAAGCTCTGC-3′; CXCL10, forward, 5′- TGGCTAGTCCTAATTGCCCTTGGT-3′; reverse, 5′-TCAGGACCATGGCTTGACCATCAT-3′,. Real-time PCR was performed using the iQ5 real-time PCR Detection System (Bio-Rad, Hercules, CA) in total 25 μl reaction mixture with 12.5 µl SYBR Green qPCR Master Mix (2×) (Bio-Rad, Hercules, CA) and 300 nM primer. The reaction conditions were as follows: 10 min at 95 °C, followed by 40 cycles of 15 s at 95 °C and 60 s at 60 °C. The HPRT (*Hypoxine* Phosphoribosyl- Transferase) forward primer (5′-CCCTCTGGTAGATTGTCGCTTA-3′) and reverse primer (5′-AGATGCTGTTACTGATAGGAAATTGA-3′) were used as an endogenous control. Quantified results represent the fold induction of target gene expression at different days post infection in comparison to the target gene expression in naïve cDNA samples. Analysis on 1.5% agarose gels was performed to exclude nonspecific amplification. NTC, no-template control (reagent alone without template) was included in each assay to detect any possible contamination of the PCR reagents. Absolute copy numbers of SPARC, CCL21, CCL19, and CXCL10 were determined using a standard curve relative to the reference gene HPRT^35^. Parasite burden was measured by amplifying the *T. gondii* gene B1 by real-time PCR as previously described^35,39,40^.

### In situ* hybridization*

Mice were terminally anesthetized with halothane and subjected to intracardiac perfusion with 4% paraformaldehyde. Brain tissue was post-fixed in 4% paraformaldehyde and 30% sucrose solution prior to cryosectioning. In situ hybridization was performed on free-floating 25 ml cryosections as previously described^41,42^. In brief, sagittal sections were hybridized at 55 °C for 16 h with ^33^P-labeled SPARC riboprobes (10^7^ cpm/ml; accession # X04017.1, nucleotides 1601-2036). Excess probe was removed by washing at room temperature (23 °C) for 30 min in 0.03 M NaCl, 0.003 M sodium citrate (2 × SSC) containing 10 ul β-mercaptoethanol, followed by a 60 min incubation with 4 μg/mL ribonuclease, 0.5 M NaCl, 0.5 M EDTA, 0.05 M Tris–HCl, pH 7.5, at 37 °C. Sections were then washed under high-stringency conditions for 90 min at 55 °C in 0.5 × SSC, 50% formamide and 10 um β-mercaptoethanol, followed by a 60 min incubation at 68 °C in 0.1 × SSC, 5 ul β-mercaptoethanol and 0.1% N-lauryl sarcosine. Sections were subsequently mounted onto Fisherbrand superfrost plus slides and dehydrated with ethanol and chloroform. Slides were exposed for 3 days to Kodak X-AR film.

### Astrocyte cultures

Within three days of birth, brains were dissected from newborn mice and pressed through a 40 μm cell strainer with 20 mL 2% FBS (fetal bovine serum) in DMEM and spun at 2000 rpm for 10 min. The cell pellet was washed and spun twice more to remove myelin, resuspended in complete DMEM (DMEM, 10% FBS, 1% non-essential amino acids, 2 mM glutamine, 50 IU/mL penicillin, 50 mg/mL streptomycin, and 10 mM Hepes) and plated in 25cm^2^ flasks. Every other day for the next 6 days cultures were fed with fresh complete DMEM. After two more days, flasks were shaken for 2 h at 260 rpm at 37 °C. Media was replaced with fresh complete DMEM and flasks were shaken at 100 rpm for an additional 24 h. Cells were detached with trypsin–EDTA (Corning, Glendale, AZ), resuspended in complete DMEM at 1 × 10^6^ cells/mL and plated in 24 well plates. The following day, cultures were stimulated with media alone, 200U/mL IFNγ, 200 μg/mL soluble *T. gondii* antigen (sTAg), or infected with type I (RH) or type II (Pru) parasites at an MOI of 3:1. Astrocytes were stimulated for 24 h before harvest for RTqPCR or immunohistochemistry.

### Immunohistochemistry

Immediately following excision, brains were bisected sagittally and flash-frozen in isopentane. Frozen brains were embedded in Optimal Cutting Temperature (OCT) solution (Tissue-Tek, Torrance, CA) put on dry ice and subsequently stored at -80 °C. Serial sections of 10–20 μm were prepared on a standard Cryostat machine (LEICA/CM1850, Simi Valley, CA). Frozen tissue sections or astrocyte cultures were fixed 75% acetone/25% ethanol then blocked in 2–10% donkey serum (Jackson Labs) prior to incubation with purified primary antibodies. Antibodies for SPARC (Abcam, Cambridge, MA or R&D Systems, Minneapolis, MN), CCL21 (Peprotech, Rock Hill, NJ), or GFAP (Life Technologies, Grand Island, NY) were incubated with tissue samples for 3 h at room temperature or overnight at 4 °C, and followed with appropriate secondary antibodies conjugated to Alexa 488, Alexa 568, or Alexa 647 (Life Technologies, Grand Island, NY). Samples were mounted in Prolong Gold with DAPI (Life Technologies, Grand Island, NY) for nuclear counterstaining. Images were collected on a Leica SP2 scanning confocal microscope (Leica Optics, Germany), and analyzed using Improvision Volocity 5.0 (Perkin-Elmer, Waltham, MA).

### Gel filtration

Recombinant murine SPARC, CCL21, CCL19, CXCL10 (5ug each, R&D Systems or Peprotech) or BSA (Bovine Serum Albumin) (Sigma, St. Louis, MO) were resuspended in 70μL phosphate buffered saline (PBS) with 2 mM Calcium, mixed in the pairs indicated in Fig. 4, and filtered using Sephadex G50 at 4 °C. Protein standards composed of BSA, ovalbumin (Sigma, St. Louis, MO), trypsin (Corning, Glendale, AZ), and CCL19 were also run through the column individually. Absorbance of eluted fractions at 280 nm were recorded and fractions that contained protein were pooled and concentrated with Amicon filters (Millipore, Billerica, MA). Concentrated eluate was mixed with Laemmli buffer and subjected to SDS-PAGE on 10% Tris–HCL gels (Biorad, Hercules, CA) followed by western blot onto nitrocellulose membranes on a semi-dry blotter (Biorad, Hercules, CA). Membranes were blocked with 5% nonfat dry milk then probed with anti-SPARC (R&D Systems), anti-CCL21 (Peprotech), anti-CCL19 (R&D Systems) or anti-CXCL10 (R&D Systems) antibodies and corresponding secondary antibodies (anti-rabbit 680 or anti-goat 800, Li-COR Biosciences). Blots were imaged using an Odyssey Infrared Imaging system (Li-COR Biosciences).

### Thermophoresis

Microscale thermophoresis analyses were carried out with a Monolith NT.115 instrument (Nano Temper, Munich, Germany)^43^. Recombinant murine SPARC was covalently linked to the fluorescent label NT-495 by NHS coupling according to the Nano Temper’s instructions. Increasing concentrations of unlabeled CCL21, CCL19, and CXCL10 in Ca-MST buffer (50 mM Tris, pH 7.4, 150 mM NaCl, 2 mM CaCl_2_, 0.05% Tween 20) were titrated against 4.5 nM of labeled SPARC. Standard treated capillaries (K002 Monolith NT.115) were loaded with the samples, and all measurements were performed three times at room temperature. Data acquisition and processing was performed with the NanoTemper’s analysis software using the thermophoresis plus t-jump method (Nano Temper, Munich, Germany), and non-linear regression was performed in Prism 6 (GraphPad, La Jolla, CA) to estimate dissociation constants according to NanoTemper fit function for K_d_^44^. Metrics for curve fitting (R^2^ and AICc) are also reported in the text.

### Chemotaxis assays

Collagen IV coated μ-Slide Chemotaxis slides (ibidi, Madison, WI) were seeded with C57Bl/6-dsRed^+^CD8^+^ T cells (500,000 cells/mL) isolated from splenocytes purified by CD8^+^ T cell enrichment columns (R&D Systems, Minneapolis, MN) suspended in 2.4 mg/mL Matrigel Matrix (Corning, Tewksbury, MA) according to the manufacturer’s instructions, in the presence or absence of SPARC (1000 ng/mL). Slide reservoirs were filled with assay media (RPMI, 5% BSA) on either side, CCL21 (1000 ng/mL) on either side, or with media on one side and CCL21 (2000 ng/mL) on the other to establish a gradient. Cells were filmed for 20 min at 37 °C, 5% CO_2_ and 20% O_2_ on a Zeiss 710 LSM confocal microscope. A minimum of 16 cells were tracked per group per experiment. Only cells that were visible in the field of view for a minimum of 5 frames were included in the analysis. Cell behavior was analyzed with Volocity 5.0 or 6.1 (Perkin-Elmer, Waltham, MA).

### Adoptive T cell transfer

Splenocytes from OTI-dsRed^+^ mice were expanded in vitro as previously described^45^. After 9 days of expansion, 5 × 10^6^ cells per mouse were injected into C57Bl/6 or SPARC−/− mice infected for 3 weeks intravenously.

### Multiphoton microscopy and SHG analysis

Fixed and stained flash-frozen tissue for SHG imaging was mounted with Prolong Gold (without DAPI, Life Technologies, Grand Island, NY) and imaged using the Leica TCS SP8 MP demo scope equipped with a 40× water objective and a Ti:Sapphire femtosecond laser (720–980 nm, < 140 fs; 90 MHz; Coherent Chameleon) tuned to 860 nm. SHG images were taken at 20 μm z stacks with a step size of 1 μm were taken, converted to extended focus and visualized using Volocity 5.0 (Perkin-Elmer, Waltham, MA).

For preparation of brain tissue for live imaging, mice were euthanized by CO_2_ asphyxiation, the brain was removed, placed in a 35 mm glass bottom dish and immediately transferred into an incubating chamber with RPMI complete (RPMI plus 10% fetal-calf serum, 1% pen/strep, 1% glutamine, 1% HEPES, 1% nonessential amino acids, and 0.1% β-mercaptoethanol), and maintained at 37 °C, 5% CO_2_. For SHG studies time course, imaging was performed on a Zeiss 710 LSM microscope equipped with a 40× (NA 0.8) water objective. The setup included external non-descanned dual-channel/fluorescence detectors and a diode-pumped, wideband mode-locked Ti:Sapphire femtosecond laser (720–980 nm, < 140 fs; 90 MHz; Coherent Chameleon). To quantify the density of SHG, we generated 30 μm z stacks with a step size of 1 μm. We used Volocity (Perkin Elmer, Waltham, MA) for all measurements. Skeletal volumes were measured using a size filter of > 50 μm and calculated from at least six z stacks from five infected C57Bl/6 and SPARC−/− mice. To visualize the extent of SHG fiber formation, C57Bl/6 and SPARC−/− mice were infected for two weeks before they were sacrificed and their brains were excised. Using a stainless steel adult mouse brain matrix (Zivic Instruments), brains were sliced in 2 mm sections. Fresh slices that were cut at approximately 1.35 mm caudal to bregma (approximately corresponding to coronal level 68 of the Allen Brain Atlas) were used for imaging. Slices were kept moist with 1× phosphate buffered saline during image acquisition. Tiled images were rendered using the Zeiss Zen software.

For in vivo cell migration studies, imaging was conducted on a Nikon FN1, similarly equipped with a Ti:Sapphire femtosecond laser (720–980 nm, < 140 fs; 90 MHz; Coherent Chameleon). C57Bl/6 and SPARC−/− mice infected with Pru-OVA for four weeks received an intravenous injection of 5 × 10^6^ dsRed^+^ OVA-specific T cells that have been activated and expanded in vitro with OVA protein and IL-2^37^. One week later, the mice were sacrificed and their brains were prepared as described^37^. Brain tissue was secured to a 35 mm petri dish with super glue such that the cortex was fully exposed to the 25× dipping objective. Tissue was perfused with oxygenated cRPMI and kept at 37 °C. All movies were acquired as a Z-stack series with a thickness of 30 μm (with a 6 μm step size) for 30 min. Samples were exposed to polarized laser light at a wavelength of 920 nm, determined to be ideal for collecting both dsRed and SHG signal. Emitted light was separated with a filter set (primary dichroic mirror, followed by dichroic mirrors at 495 nm, 520 nm, and 575 nm). Four photo multiplier tubes collected light at wavelengths: 457–487 nm; 503–537 nm; 525–570 nm, and 580–652 nm. Single cell tracking analysis was conducted automatically and manually on a minimum of 20 frames to calculate mean velocities, displacement, and meandering indices of migrating cells as previously demonstrated^37,45^.

For blood vessel imaging, sulforhodamine B (SRB, Sigma) was injected into infected mice intravenously and allowed to circulate for 30 min before mice were sacrificed and their brains were removed for imaging. Similarly to cell migration studies, tissue was excited by 920 nm light and SHG signal was collected at 457–487 nm and SRB red signal was collected at 580–652 nm.

### Statistics

For statistical analysis of survival data, the log-rank test was used. For data with a non-gaussian distribution, the Mann–Whitney test was used. For normally distributed data, an unpaired, two-tailed Student’s t test, or ANOVA test with a 95% confidence interval was used (Prism; GraphPad Software, Inc., La Jolla, CA). All data are represented as mean ± SEM.

## Results

### Parenchymal SHG signal is distinct from neurons and brain vasculature

**Figure 1:**
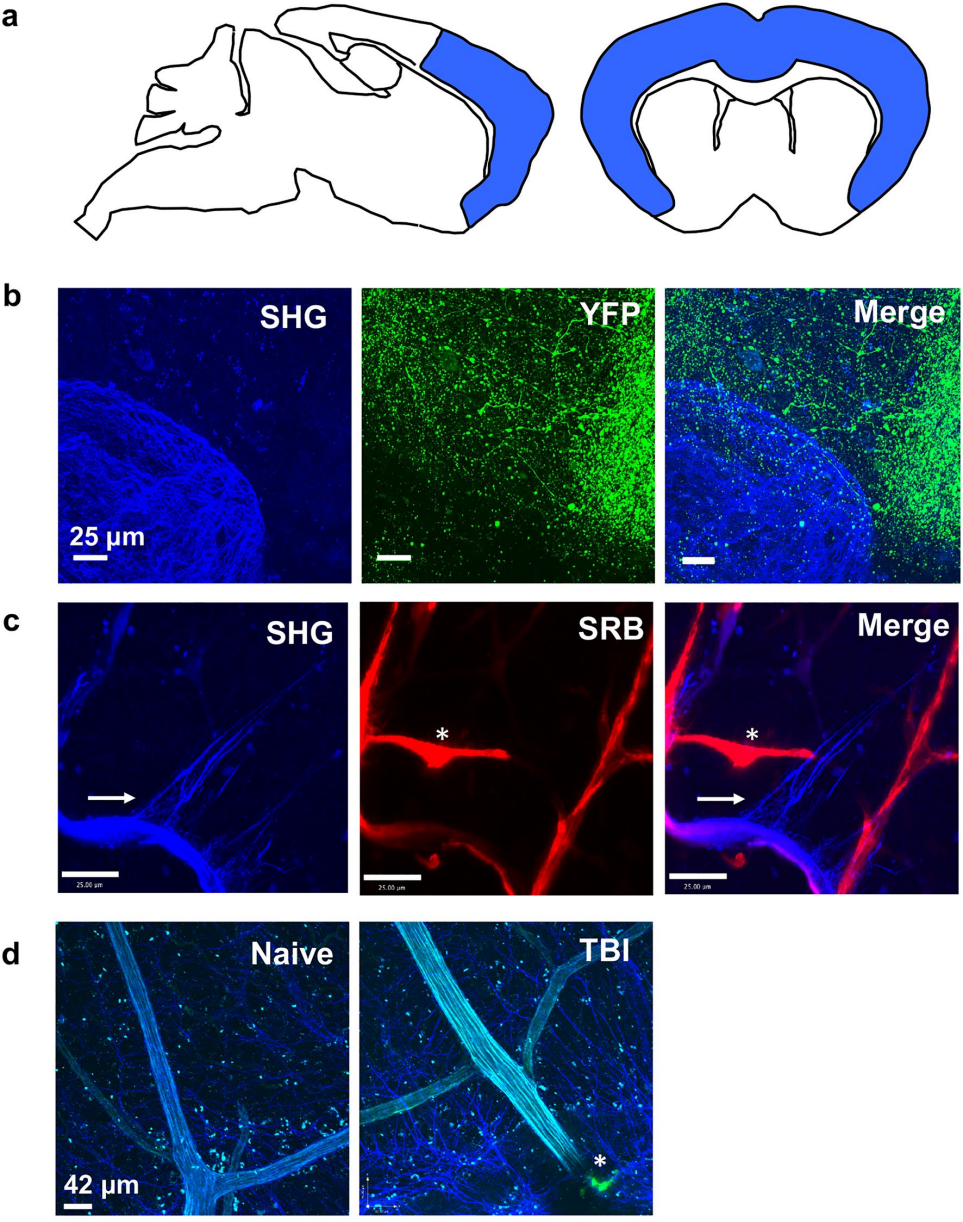
Parenchymal SHG signal is distinct from neurons and brain vasculature. (**a**) All imaging, multiphoton and confocal, focused on the frontal cortex of the mouse brain, highlighted in blue. (**b**) Thy1.1-YFP reporter mice were infected with Pru-OVA for 14 days before fresh (unfixed) brains were imaged using multiphoton microscopy to visualize fluorescent neurons (green) and SHG signal (blue). (**c**) Similarly, C57Bl/6 mice infected with Pru-OVA for 14 days were injected with the fluorescent dye SRB (red) i.v. 30 min prior to multiphoton imaging to distinguish the parenchymal SHG signal (blue) from that generated by collagen rich blood vessels. (**d**) SHG signal (blue) in naïve mice and following traumatic brain injury. Green fluorescence (*) indicates vascular leakage of FITC Dextran at injury site. For all panels, 30 μm z-stacks were collected in areas of the frontal cortex from fresh tissue explants. Images are representative of at least 2 individual experiments where n = 3 mice.

During chronic *T. gondii* infection, a network of fibers is established within the CNS to which migrating immune cells associate^37^. The network’s presence in naïve mice is minimal and therefore is theorized to require a substantial amount of tissue remodeling prior to or during chronic infection. The original study that reported the existence of inflammation-associated fibers in the brain excluded the possibility that the SHG signal emanated from reactive astrocytes^37^. To build on that work, we wanted to confirm that the fibers are indeed extracellular and not part of other abundant cells or structures in the brain. We focused our imaging in the frontal cortex since the SHG signal is in greatest abundance where *T. gondii* is also most common^30^ (Fig. 1a). We first infected Thy1.1-YFP mice (with YFP expression in neurons) and imaged various layers of cortex explants at 14 days post infection (Fig. 1b). We determined that the neuronal signal and the SHG signal induced upon infection is distinct (Fig. 1b). Next, as collagen is a known emitter of second harmonics, and the richest sources of collagen in the brain are blood vessels, we investigated whether blood vessels could be the source of SHG signal in the brain. C57Bl/6 mice were infected for 14 days, then perfused with the fluorescent dye sulforhodamine b (SRB) for 30–60 min to highlight all blood vessels prior to imaging cortical explants. As anticipated, SRB and SHG signals colocalize in-line with the presence of collagen-containing vessels (Fig. 1c). In addition, there are areas where SRB has escaped vessels consistent with previous reports of an opening of the blood brain barrier and leakage (Fig. 1c, asterisk)^46,47^. However, there are also long thin linear strands of second harmonic signals that emanate from vessel areas that are not labeled by SRB (Fig. 1c, horizontal arrow). The original report determined that parenchymal SHG signal is not unique to *T. gondii* infection as it is also present in experimental autoimmune encephalomyelitis (EAE) in both the brain and spinal cord^30^. To determine if the association with blood vessels is observed in other models of injury we imaged these fibers following the induction of traumatic brain injury (TBI) (Fig. 1d). Similar to infection, we see second harmonic signals colocalizing with blood vessels labeled with FITC dextran and an increase in signal around damaged vessels as observed with dextran leakage at the point of impact (Fig. 1d, asterisk). Therefore, together with the original report describing these structures, we are confident that the SHG-emitting fibers are extracellular, may be partially composed of reorganized extracellular matrix proteins that are present in blood vessels, and arise in the CNS in models of infection, autoimmunity, and injury.

### SPARC is upregulated in the brain during *T. gondii* infection

**Figure 2:**
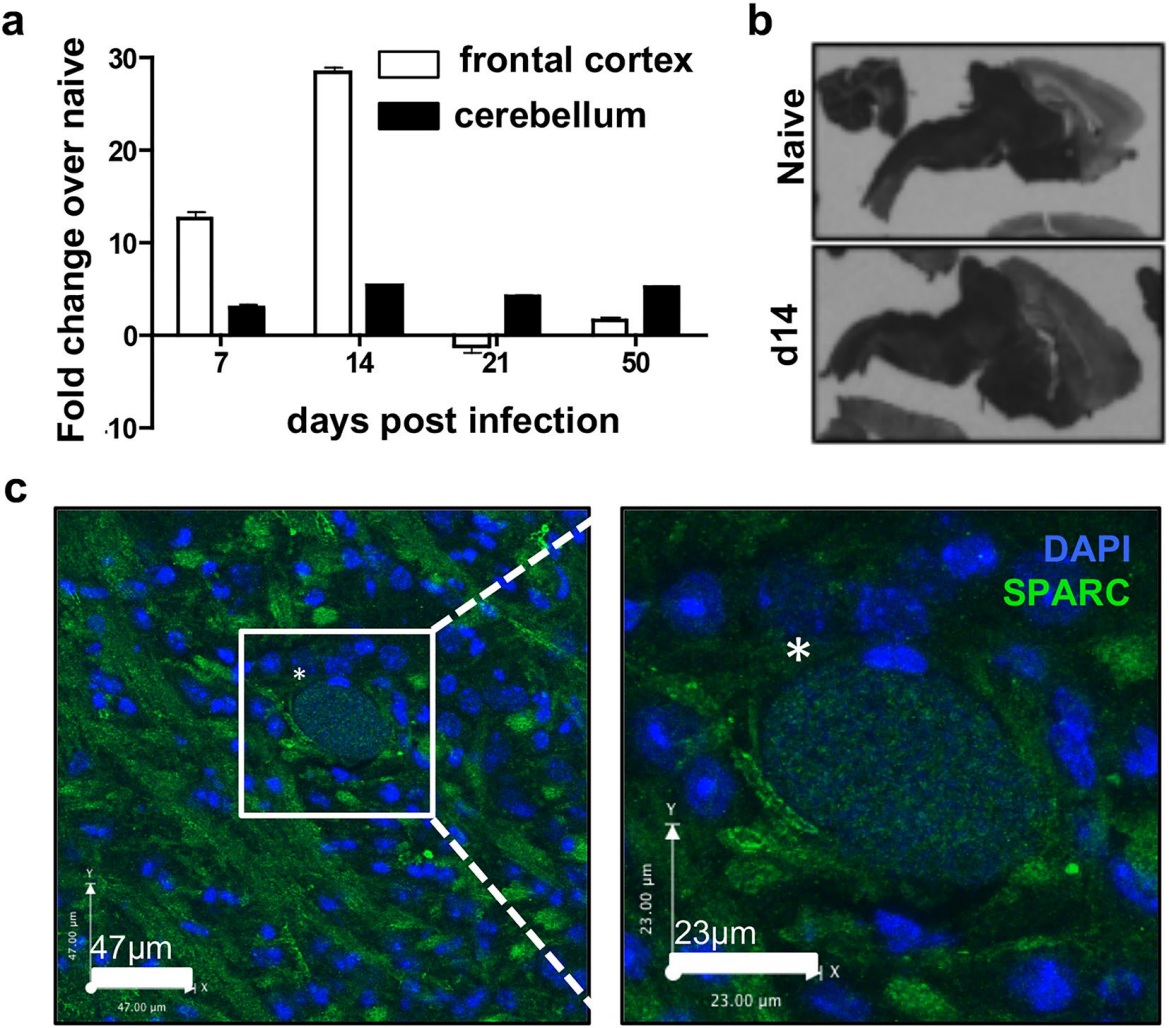
Expression of SPARC in the CNS during chronic *T. gondii* infection. (**a**–**c**) C57Bl/6 mice were infected with the Me49 strain of *T. gondii* and sacrificed at various timepoints. (**a**) Frontal cortex and cerebellum of infected brains were removed at days 7, 14, 21, and 50 post-infection. RNA was isolated, reverse transcribed and analyzed for levels of SPARC transcripts using qRT-PCR. Results are shown as fold change over naïve normalized to HPRT. n = 3 mice per group. Data are represented as mean ± SEM. (**b**) Brains were removed from naïve and 14 day infected mice. In-situ hybridization was performed on brain slices with probes specific to SPARC mRNA. (**c**) Confocal fluorescence microscopy of 20 μm brain slices taken from mice at 3 weeks post infection. Image and inset show a parasite cyst (*) identified by punctate DAPI (blue) stain surrounded by parenchymal SPARC (green).

Since SPARC is a molecule that is consistently associated with tissue remodeling^48^ and with cell migration^17,49^, we examined its expression profile in the CNS following infection. SPARC is known to be constitutively expressed in the cerebellum in the adult brain but is normally absent from the cortex^50^. Mice were sacrificed at various time points post infection and frontal cortex and cerebellum were analyzed for SPARC expression using RT-qPCR. SPARC expression, although slightly increased over naïve mice, was relatively stable in the cerebellum across early acute and late chronic infection (Fig. 2a). In contrast, a significant increase in SPARC mRNA occurs in the frontal cortex, an area of the brain associated with parasite infection and inflammation, beginning at day 7 and peaking at day 14 post-infection (Fig. 2a). To confirm these results, we used in situ hybridization for SPARC mRNA on brain slices taken from naïve and infected mice (Fig. 1b). Here, our results confirm heavy SPARC expression in the hindbrain, an area not normally colonized by *T. gondii*, of both naïve and 14 day infected mice and a substantial upregulation of SPARC in the frontal cortex at day 14 (Fig. 1b).

Next, we examined the localization of SPARC protein in the frontal cortex of the infected brain by immunofluorescence. In line with expectations, immunohistochemistry reveals parasite cysts in the frontal cortex (Fig. 1c, asterisk). These cysts are the source of lifelong infection and exist intracellularly within neurons. They can reach up to 80 μm in diameter, are distinctively round and can be identified by the punctate appearance of DAPI labeling individual parasite nuclei (Fig. 2c, inset). In contrast, SPARC appears as cellular and possibly acellular structures closely associated with cysts (Fig. 1c). Together, these data demonstrate SPARC upregulation in the CNS as a result of parasite infection. Furthermore, this increase in SPARC occurs in areas of the brain that are associated with parasite replication and inflammation.

### Reticular fibers in the CNS are compromised in SPARC-null mice following infection

**Figure 3:**
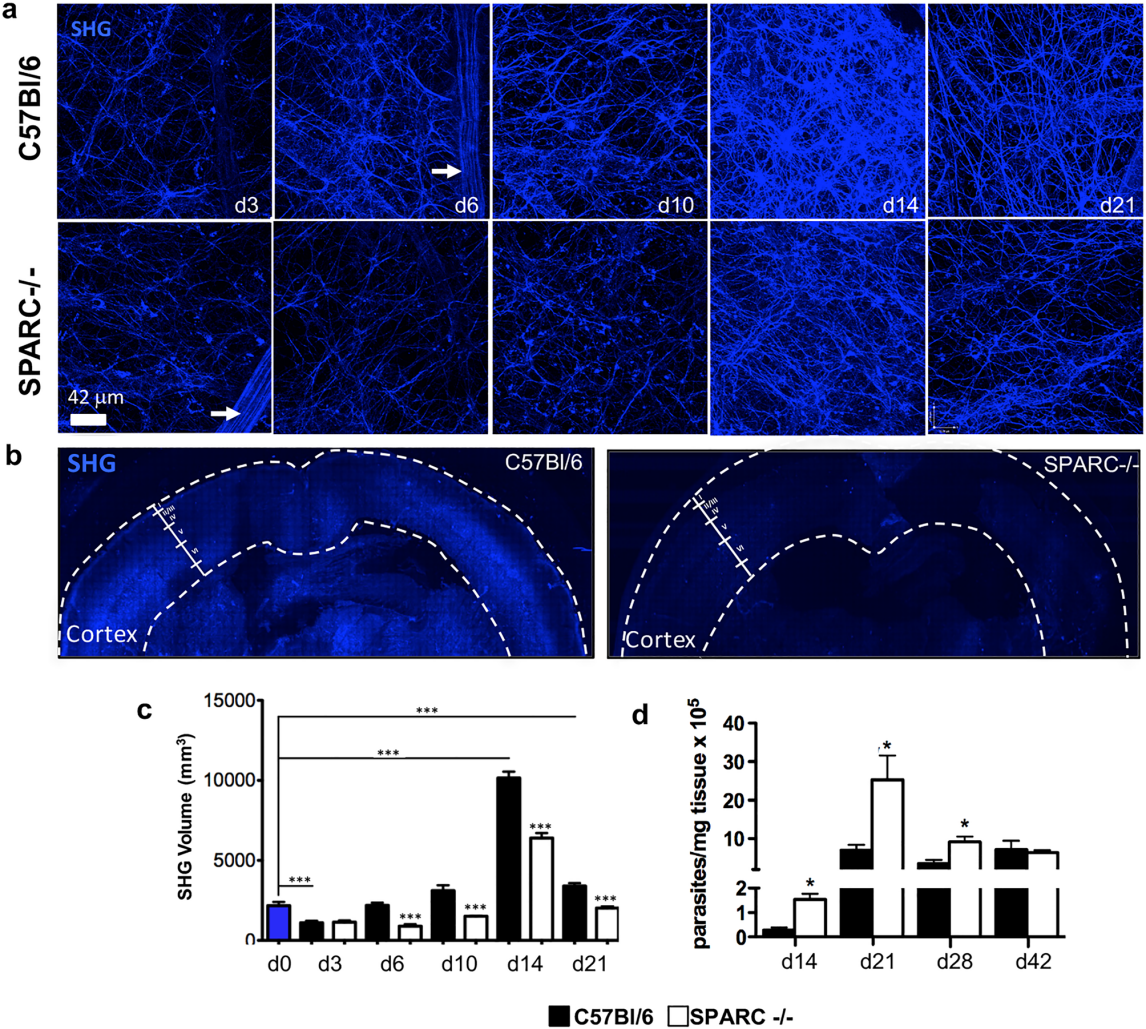
SPARC−/− mice have compromised reticular fiber network in the brain during chronic *T. gondii* infection. C57Bl/6 and SPARC−/− mice were infected and sacrificed at 3, 6, 10, 14, and 21 days following infection. Brains were removed and imaged on a multiphoton microscope for second harmonic generation (SHG) signal. (**a**) 30 μm z-stacks were obtained at various locations of the cortex. 3D images were compiled and reticular fibers were analyzed for volume (**c**). Blood vessels (white arrows) were excluded from analysis. (**b**) Brains were excised from mice at 14 days post infection and images of whole coronal sections were generated by stitching overlapping Z stacks collected over the entire section. (**d**) C57Bl/6 and SPARC−/− mice were infected and sacrificed at 14, 21, 28, and 42 days following infection. DNA was isolated from the brain and analyzed for parasite burden using qPCR. Results are displayed as parasites per mg tissue. Data are representative of at least 2 individual experiments with a minimum of n = 4 mice per group and are represented as mean ± SEM. Volumes and parasite burden were analyzed using the student’s T test, ***p < 0.001.

Fiber networks that facilitate cell migration have been observed in the tumor microenvironment as well as in inflamed tissue following infection or insult^10,37,51^. The extent to which SPARC regulates the assembly of these networks is currently unknown, however studies have drawn a correlation between SPARC-cell interactions with the ECM leading to efficient migration^28,52,53^. To examine the role of SPARC in the creation or maintenance of the network in the CNS following infection, C57Bl/6 and SPARC−/− mice were infected with *T. gondii* and imaged for SHG within the brain. To determine the kinetics of this tissue remodeling, multiple 30 μm z-stacks were collected from cortical layer I over the course of infection at days 3, 6, 10, 14, and 21 following infection (Fig. 3a). Visualization of the second harmonic signal shows two types of structures in C57Bl/6 mice. First, a strong dense signal that highlights blood vessels as previously described^37^ (Fig. 3a, arrow), likely derived from collagen, was not included in volumetric analysis. Secondly, thin (< 1 μm) web-like strands were observed that are sparse early following infection and increase in density until reaching an entangled appearance at 14 days post infection (Fig. 3a). By day 21 fibers are reduced but take on a more linear, organized appearance. In contrast to C57Bl/6 mice, the second harmonic signal in the brains of SPARC-null mice is significantly diminished (Fig. 3a). Images show a reduced density and linearity in the signal, especially at later time points. To determine the extent to which SHG fibers penetrate the brain, C57Bl/6 and SPARC-deficient mice were sacrificed at 14 days post infection, peak SHG volume, and their brains were removed and sliced coronally. Fresh, whole coronal slices cut at approximately 1.36 mm caudal to bregma were subjected to two-photon microscopy and SHG signal was collected in tiles across the entire section and stitched (Fig. 3b). In C57Bl/6 mice, imaging reveals SHG in substantial areas of the cortex, specifically a distinct signal in layer I, reduced signal in layers II/III/IV and a strong signal from layers V and VI (Fig. 3b) as well as around the ventricles. Images of SPARC-deficient mice show a dramatic reduction in the overall SHG signal with no definition of cortical layers (Fig. 3b). Volumetric quantification of the production of the second harmonic signal over time (Fig. 3c) confirms SHG volume in C57Bl/6 mice peaks at day 14 and SHG volume is significantly decreased in the brains of SPARC−/− mice as compared to C57Bl/6 at nearly every timepoint from early acute to early chronic infection (Fig. 3c).

To examine whether the kinetics of network formation correlate with parasite burden in the brain, C57Bl/6 and SPARC−/− mice were sacrificed at various stages of infection. Brains were harvested and DNA was isolated for analysis of parasite burden by RTqPCR relative to a standard curve (Fig. 3d). Analysis of parasite burden reveals parasite DNA is significantly elevated in the brains of SPARC−/− mice compared to C57Bl/6 beginning at day 14 following infection (Fig. 3d) a difference not observed in peripheral organs during acute infection (Fig. S1). Parasite burden for both C57Bl/6 and SPARC−/− mice reach a maximum at day 21 post infection. In C57Bl/6 mice, parasite burden remains consistent 21 through 42 days post infection. By comparison, parasite burden in the SPARC−/− brain is significantly elevated compared to C57Bl/6 mice at day 21, remains elevated compared to C57Bl/6 mice at day 28, though well below its peak at day 21, then is reduced to the level of C57Bl/6 mice by day 42 post infection. These data suggest that tissue remodeling occurs during a time when the parasites first disseminate to the brain. Further, SPARC deficiency leads to an earlier dissemination to, or poorly controlled replication of the parasite in the brain resulting in increased parasite burden during early chronic infection, which is then resolved as chronic infection progresses.

### SPARC binds CCR7 ligands

**Figure 4:**
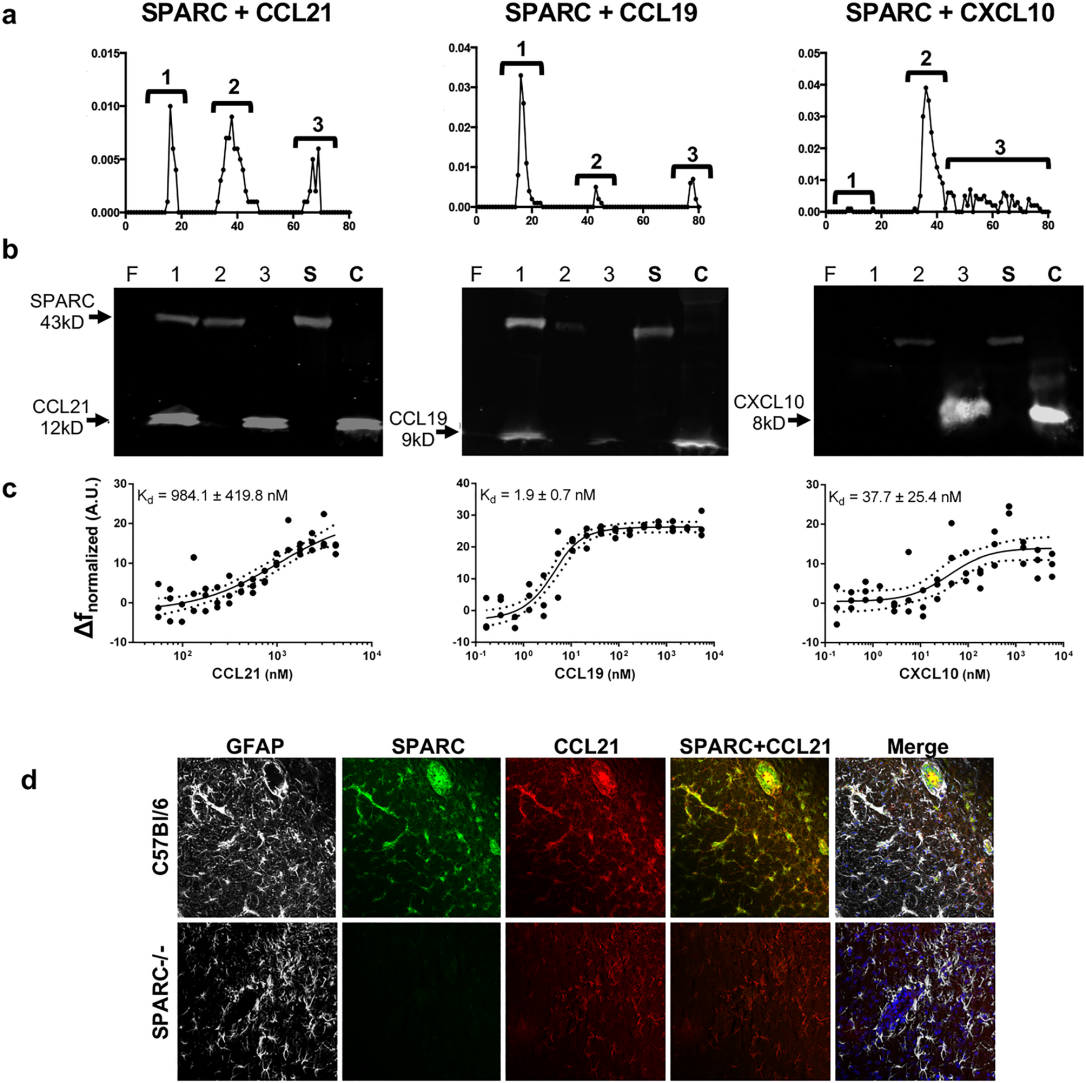
SPARC interacts directly with CCR7 ligands. Recombinant SPARC was incubated with CCL21, CCL19, CXCL10, or BSA (shown in Supplementary Fig. S2) and the mixture and applied to a Sephadex G-50 column. (**a**) The elution profiles (SPARC + CCL21; SPARC + CCL19; SPARC + CXCL10) are annotated to show which fractions were pooled and subjected to western blot. (**b**) Results from western Blots (SPARC + CCL21; SPARC + CCL19; SPARC + CXCL10) are shown under their respective elution profiles. (**c**) Thermophoretic plots (SPARC + CCL21; SPARC + CCL19; SPARC + CXCL10) are shown under their respective western blots. Values presented are the calculated dissociation constants ± SEM. The y-axis illustrates the thermophoretic response as observed by a change in normalized fluorescence in arbitrary units. Dotted lines illustrate the 95% confidence interval for the fit from non-linear regression. (**d**) Immunohistochemistry of chronically infected C56Bl/6 and SPARC−/− mice were incubated with antibodies to GFAP (white), SPARC (green), and CCL21 (red).

SPARC is well known to facilitate ECM remodeling by binding to matrix components^48^. Many matrix components are thought to be structural in nature to provide a scaffold for tissue growth and cell migration. However, chemokines are also secreted into the extracellular space and may be subject to the same remodeling mechanisms as matrix constituents. Specifically, CCL21, upregulated in the brain along with CCL19 and CXCL10 following *T. gondii* infection, is known in other models to bind matrix molecules through its basic C-terminal domain and has been theorized to prompt haptotaxis, migration induced in tissue by an immobilized chemokine gradient^54^. Indeed, as SPARC’s N-terminal domain is acidic, SPARC and CCL21 might interact directly, either to facilitate remodeling within the extracellular space or to present CCL21 in the correct confirmation to elicit movement of infiltrating T cells. To test if these proteins interact, recombinant SPARC and CCL21 (5 μg each) were mixed and applied to a 50 mL size exclusion column packed with Sephadex G-50. The absorbance at 280 nm of each 2 mL fraction eluted from the column was plotted on an elution profile (Fig. 4a). Three peaks appear on the elution profile, indicating that some of the proteins applied to the column elute together as a complex. To confirm this finding, the fractions that contained protein were concentrated and subjected to western blot. Both SPARC and CCL21 are detected in the first peak. The remaining SPARC elutes in the second peak and the remaining CCL21 elutes in the third peak (Fig. 4b). To independently verify the interaction and to quantify the affinity SPARC has for CCL21, microscale thermophoresis (MST) was used to calculate the dissociation constant. MST allows quantification of binding events as molecules move in a temperature gradient^55^. CCL21 was diluted using a 3:1 serial dilution ratio and titrated against SPARC. Using this method, the dissociation constant was calculated to be 984.1 ± 419.8 nM (AICc = 124.5, R^2^ = 0.768) (Fig. 4c), a value similar to SPARC’s known interaction with collagen (1–2 μM)^56^, but a much weaker interaction than CCL21′s known interaction with collagen (4.6 nM)^57^. To test if SPARC binds CCL21 through its basic C terminus, CCL19 was also tested. Like CCL21, CCL19 binds to CCR7 to elicit cell migration, but unlike CCL21, it lacks a C-terminal tail and consequently remains a soluble chemokine even when expressed in tissue^58^. When gel filtration is repeated with recombinant CCL19, three distinct peaks are seen in the elution profile (Fig. 4a). Surprisingly, more protein eluted in the first peak than in the other peaks in the profile. Western blotting confirmed the majority of SPARC and CCL19 applied to the column elutes as a complex (Fig. 4b). Thermophoretic analysis using a 1:1 serial dilution ratio determined SPARC has a much higher affinity for CCL19 than CCL21 (1.9 ± 0.7 nM, AICc = 135.3, R^2^ = 0.901) (Fig. 4c). Some dependence of fluorescence intensity on the concentration of CCL19 was observed within the region of the dose–response curve where changes in concentration of the ligand result in changes in normalized fluorescence. However, removal of those data points that differ more than 15% from the average change in normalized fluorescence did not significantly change the calculated dissociation constant. All data points are retained in the calculation of the dissociation constant. To determine if SPARC binding is specific for CCR7 ligands versus an ability to bind multiple small chemokine proteins, SPARC’s capacity to bind CXCL10 was also tested. CXCL10 is another chemokine known to have a major influence on the migratory behavior of infiltrating T cells in the brain during *T. gondii* infection^34^. Repeating the gel filtration with CXCL10 reveals no peak where a protein complex should elute and a distinct peak where SPARC elutes (Fig. 4a). CXCL10 does not elute in a single peak, as the chemokine forms dimers and tetramers in solution^59^. Fractions where a protein complex should have eluted from the column were concentrated along with fractions that contained protein. Western blotting of fraction concentrates confirmed no protein was present where a complex should elute and all SPARC and CXCL10 eluted separately (Fig. 4b).Thermophoresis estimates a dissociation constant of 37.7 ± 25.4 nM (Fig. 4c). However, the standard error of this measurement is nearly as large as the dissociation constant itself, and the fit is poor (AICc = 162.2, R^2^ = 0.528) compared to CCL19 and CCL21 (Fig. 4c). As a negative control to confirm that SPARC isn’t just sticky, SPARC was also run through the column in the presence of bovine serum albumin (BSA) (Supplementary Fig. 2). As expected, two distinct peaks eluted from the column in the fractions anticipated based on the molecular weight of each protein. To further confirm that proteins and protein complexes elute from the column as expected based on their molecular weights, individual proteins with distinct molecular weights were run through the column. BSA (66 kDa), Ovalbumin (45 kDa), Trypsin (23 kDa), and CCL19 (9 kDa) all eluted from the column in the fractions expected due to their size (Fig. S2). These data indicate that SPARC binds CCR7 ligands, however it is unlikely to bind CXCL10. To determine if SPARC and CCL21 can be observed interacting within the brain, immunohistochemistry was performed using SPARC-deficinet mice as a control (Fig. 4d). Significant colocalization of SPARC and CCL21 is observed especially in areas outlining vasculature. Such patterns are not observed in SPARC-deficient mice. GFAP co-staining supports astrocytes as being a dominant source for both molecules (Fig. 4d and Fig. S3).

### SPARC facilitates T cell migration in vitro

**Figure 5:**
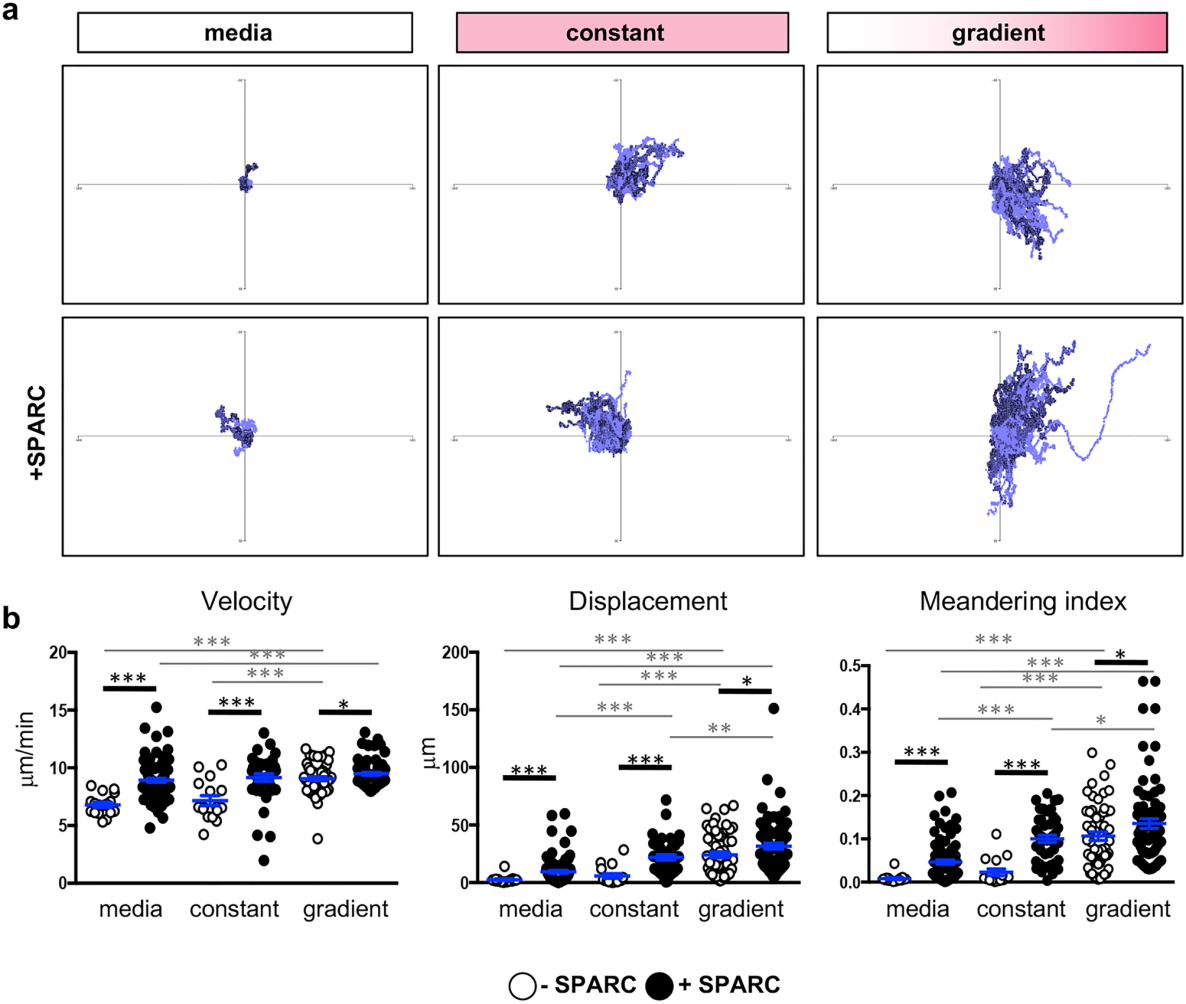
T cell migration is enhanced in the presence of SPARC in vitro. dsRed^+^ T cells were seeded in matrigel in the presence or absence of SPARC and the presence or absence of recombinant CCL21. Cell migration was imaged over 20 min in response to no chemokine, a constant concentration of chemokine, or a chemokine gradient. Videos of cell migration were analyzed. (**a**) Tracks of T cells migrating in response to media alone, a constant chemokine field, or in response to a gradient of CCL21 were normalized to begin at the origin to visualize migration trends. (**b**) Cell velocity, displacement, and meandering index were quantified using Volocity software and migration data was analyzed using the Mann–Whitney test. Data are representative of at least 2 individual experiments and are represented as mean ± SEM. Thick black lines indicate comparisons + / − SPARC, thin grey lines indicate comparison + / − chemokine. To simplify, only significant comparisons are annotated *p < 0.05, ***p < 0.001.

Both the properties of SPARC and those of CCL21 can facilitate cell migration^60,61^. However, the finding that SPARC has the ability to bind CCL21 could modulate this activity either by further increasing cell motility additively or synergistically, or by inhibiting motility by masking binding sites of either protein. To test the potential role of SPARC and CCL21 in cell trafficking within infected tissue an in vitro migration model was used where CD8^+^dsRed^+^ T cells were suspended in an artificial matrix containing collagen with or without recombinant SPARC. T cell migration was imaged in response to no chemokine, a constant concentration of CCL21, or a gradient of CCL21. Tracking of cell behavior (velocity, displacement and meandering index) reveals in the absence of a gradient, the addition of CCL21 caused no significant change in the velocity of cells with an average speed of 7 μm/min in media or CCL21 alone (Fig. 5). It is only when CCL21 is presented as a gradient that a significant change in behavior is noted. However, the addition of SPARC within the matrix significantly increases migration for all parameters (Fig. 5). The presence of SPARC increased the displacement and meandering index of the cells in the presence of CCL21, indicating they travel farther and in a more directed manner as a population. When CCL21 is added as a gradient, chemokine-dependent cell migration is at its peak with cells migrating faster, farther, and more directed than when CCL21 is present in a constant concentration across the imaging field. The addition of SPARC further enhances this effect (Fig. 5b). The greatest impact is an increase in directed migration as measured by the meandering index. To summarize, the presence of SPARC significantly enhances baseline and chemokine-dependent cell migration.

### CCL21 does not coat the fibrous reticulum that forms during brain inflammation

**Figure 6:**
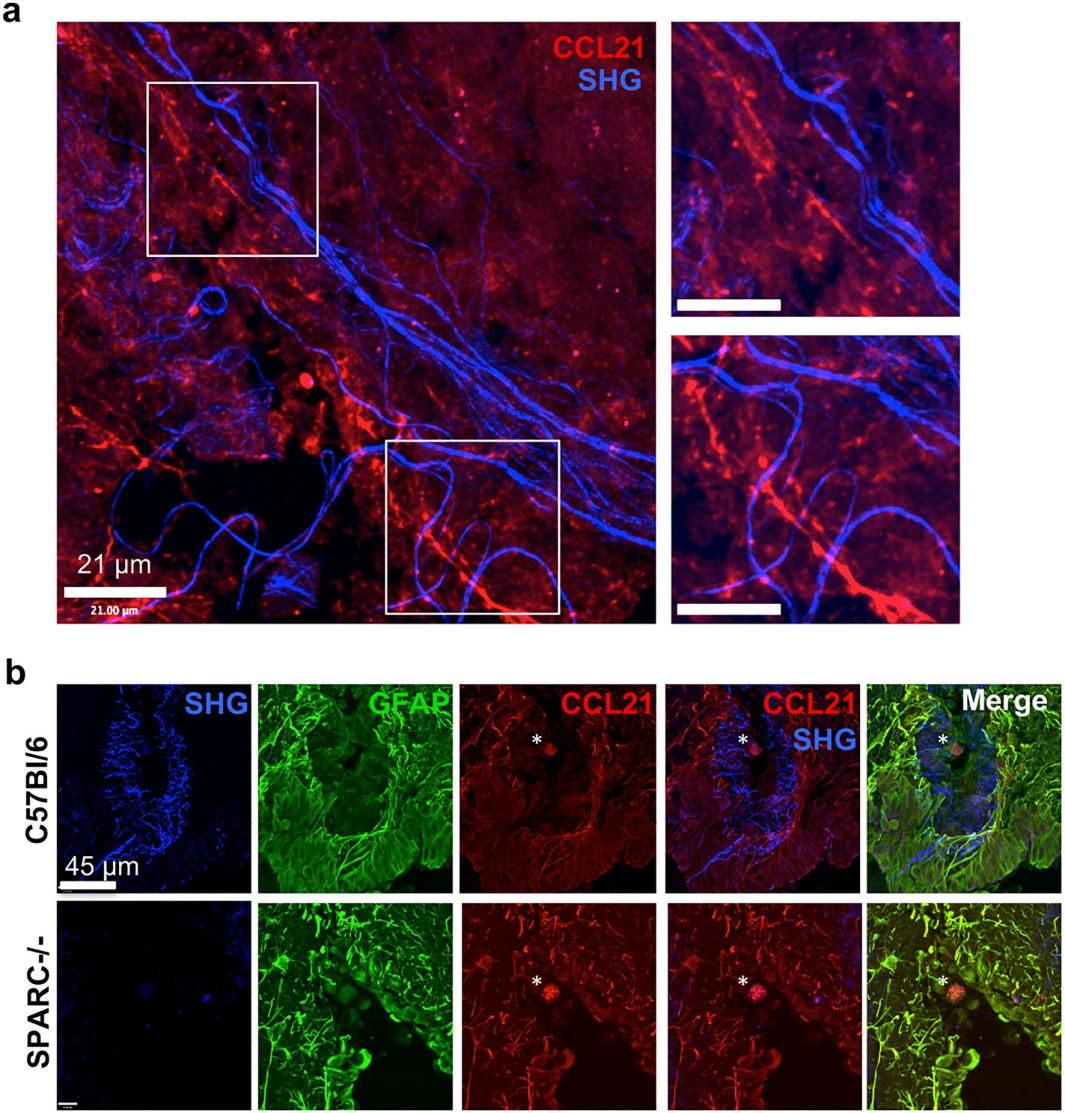
CCL21 and SHG are distinct. Flash frozen C57Bl/6 and SPARC−/− tissue sections from mice infected for 21 days were stained and imaged using multiphoton microscopy to visualize second harmonic signals found in layers 5/6 in the cortex. (**a**) CCL21 (red) SHG (blue). Insets depict SHG parallel to (top) or wrapped around (bottom) strands of CCL21. (**b**) CCL21-stained sections were counterstained with GFAP. Images are representative of 2 individual experiments where n = 3 mice per group.

Like secreted SPARC (Fig. 1c), previous immunohistochemistry results reveal strands of CCL21 in the *T. gondii*-infected brain^37^. Therefore, we hypothesized CCL21 might bind to the SHG network, perhaps facilitated by SPARC, to help coordinate T cell migration in the inflamed brain. To determine if CCL21 is bound to the network of structures with second harmonic signatures, multi-photon imaging was conducted. Visualizing the reticulum in preserved stained tissue sections is technically challenging as fixation abolishes the fine linear strands of SHG signal (data not shown). However, when tissue is flash frozen, the SHG signal is somewhat maintained allowing further probing with antibodies. Incubating sections with anti-CCL21 antibody and imaging with two-photon microscopy reveals areas where both SHG and CCL21 fibers are present (Fig. 6a), consistent with previously published results^37^, however, it does not support colocalization. Indeed, fibers emitting SHG signal seem to exist in parallel to or loosely wrapped around CCL21 (Fig. 6a, insets). Additional incubation with GFAP antibodies confirms astrocytes as the cellular source of CCL21 in the inflamed brain and that the majority of chemokine remains in contact with astrocytes, consistent with the nature of CCL21 to remain membrane bound (Fig. 6b)^58^. In these images, a *T.gondii* cyst can be seen at the center (Fig. 6b, asterisk), surrounded by structures revealed by second harmonic generation. Consistent with the original work describing the network^37^ and Fig. 6a, the second harmonic signal is distinct from reactive astrocytes. Using this method we are able to observe CCL21 staining is entirely confined to areas of reactive astrocytes and not bound by the network (Fig. 6b). Analysis of CCL21 in SPARC−/− mice reveals CCL21 in the same astrocytic location as in C57Bl/6 mice despite little SHG signal surrounding parasitic cysts (Fig. 6b). Astrocytes are thought to be the primary producers of chemokines in the CNS following infection with *T. gondii*^62–65^ and could be a result of direct infection and/or stimulation by the inflammatory milieu. At the peak of acute infection, systemic concentrations of circulating cytokines activate astrocytes, before the parasite is thought to enter the brain^62^. To test if exposure to inflammatory cytokines can trigger SPARC or CCL21 induction, primary murine astrocytes were cultured with media alone or with IFNγ, a cytokine required for control of intracellular pathogens. RNA was isolated from astrocyte cultures and tested for the production of CCL21 and SPARC transcripts relative to a standard curve. This reveals that stimulation with IFNγ cytokine alone is not sufficient to significantly induce the production of either SPARC or CCL21 (Fig. S3). In contrast, significant upregulation of both SPARC and CCL21 mRNA was observed after infection with live parasites (Fig. S3). Therefore, astrocytes that interact with live parasites, or possible parasite proteins (sTAg), can produce SPARC and CCL21 during *T. gondii* infection.

Immunohistochemistry of sections from naïve and infected brains also support astrocytes as the source of SPARC and CCL21 (Fig. S3). In situ, low levels of constitutive SPARC and CCL21 expression have been reported in the CNS^66^. In our hands little to no SPARC or CCL21 expression is detected in the cortex of naïve brains and staining for GFAP is dim (Fig. S3). In chronically infected brains, and consistent with previous reports^37,63^, GFAP is upregulated in the cortex, indicative of the morphological changes astrocytes undergo in response to CNS pathology. SPARC and CCL21 co-localize with reactive astrocytes (Fig. S3), confirming that astrocytes are the primary source of this chemokine and SPARC during *T. gondii* infection. This conclusion holds true in the absence of SPARC, as CCL21 staining is also restricted to astrocytes in SPARC−/− mice (Fig. 4d). Altogether, these data support infection-induced production of SPARC and CCL21 by astrocytes, but that neither SPARC nor CCL21 coat the fibrous SHG network.

### Reticular fibers formed upon infection facilitate efficient T cell entry into the brain

**Figure 7:**
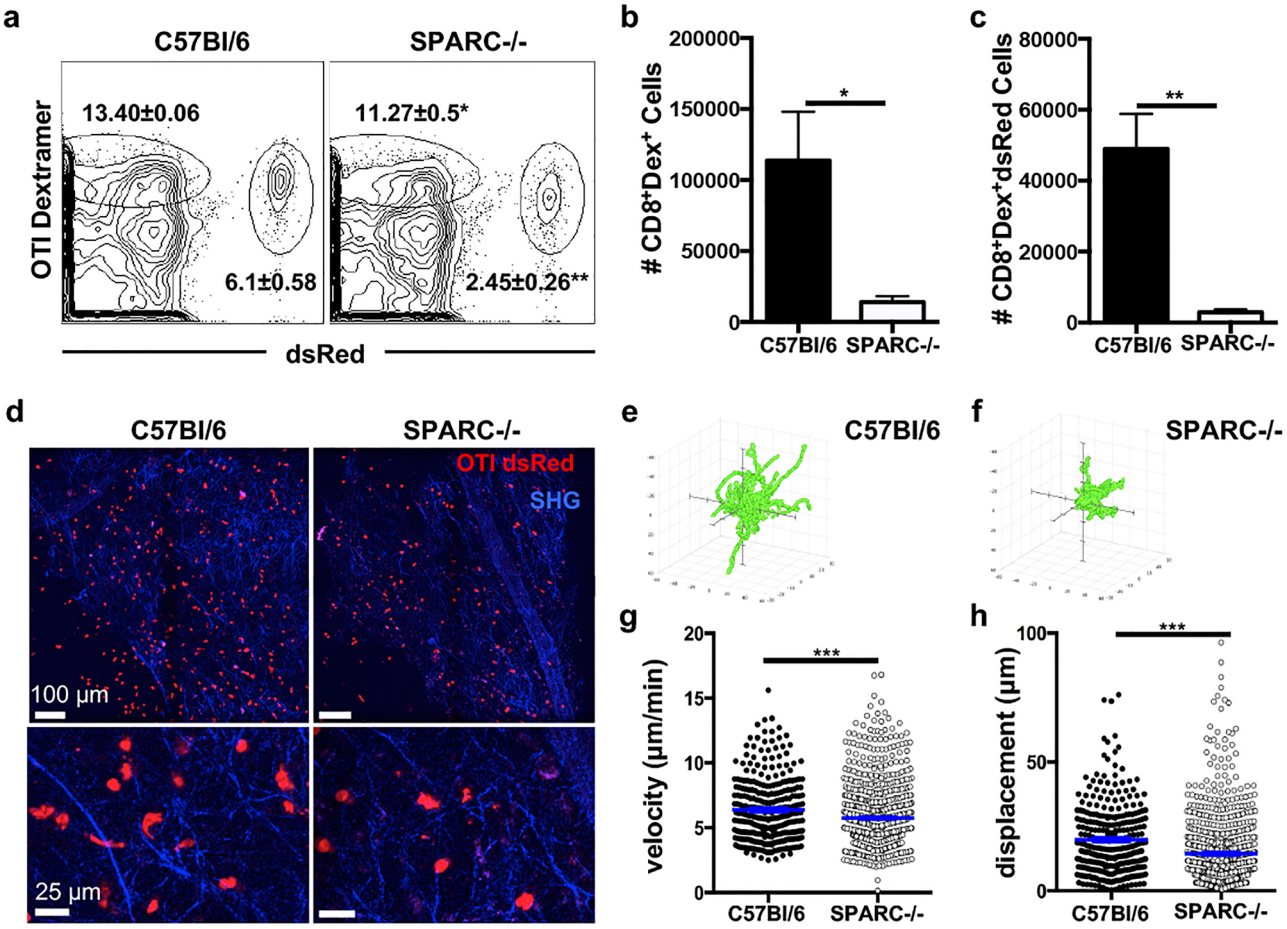
SPARC facilitates optimal CD8 + T cell migration in the brain. C57Bl/6 and SPARC−/− mice were infected with Pru-OVA for 21 days before receiving 5 × 10^6^ OVA specific dsRed^+^CD8^+^ T cells. Analyses were conducted one week post transfer. (a) Flow cytometry plots of endogenous (left gate) and transferred OVA specific dsRed T cells (right gate) isolated from the whole brain of chronically infected C57Bl/6 and SPARC−/− mice. (**b**) Absolute cell numbers of endogenous OVA specific T cells calculated from the frequencies presented in (**a**). (**c**) Absolute cell numbers of transferred dsRed^+^ OVA-specific T cells calculated from the frequencies presented in a. (**d**) 2 × 2 tiled images were taken of the cortex using two-photon microscopy to visualize the distribution of T cells infiltrating the brain (**e–h**) Videos imaged of cell migration in the infected brain were analyzed. Flower plots of cell tracks from C57Bl/6 (**e**) and SPARC−/− (**f**) normalized to the origin. Velocity (**g**) and displacement (**h**) for cells recruited to C57Bl/6 brains compared to cells migrating in SPARC−/− brains were quantified. Cells were tracked using Volocity software and migration data was analyzed using the Mann–Whitney test, ***p < 0.001. Flow cytometry data was analyzed using the student’s T test, *p < 0.05, **p < 0.01. Data are representative of at least 2 individual experiments with a minimum of n = 3 and are represented as mean ± SEM.

Reticular ECM structures that support cell migration have been observed in a variety of inflammatory models^10,37,51^ and defects in SPARC lead to altered immune responses^15,27^. Although a possible delay in CD4^+^ T cell recruitment to the site of infection was noted we could not detect any striking differences in the peripheral or CNS (Fig. S1 and S4 respectively) immune response between C56Bl/6 and SPARC−/− mice including T cell activation or cytokine production. Furthermore, the absence of SPARC did not affect the ability of mice to survive the infection (data not shown). However, it was still possible that a poorly assembled network hindered T cell migration behavior enough to explain the inability to control parasite burden in the brain at early chronic timepoints (Fig. 3d). In order to determine if the absence of SPARC and a deformed network affects T cell infiltration into the brain, C57Bl/6 and SPARC−/− mice were infected with parasites genetically engineered to produce ovalbumin (PruOVA) to allow the tracking of antigen specific T cells. Twenty-one days after infection, mice received OVA specific, activated red fluorescing (dsRed^+^) CD8^+^ T cells^37,45^ (OTI-dsRed). After one week, T cell migration was analyzed using multiphoton microscopy. This timepoint was chosen to maximize T cell infiltration in the brain while still imaging during a time when there is a significant reduction in the volume of the fibrous network in the absence of SPARC. Analysis of T cell populations in the brain reveals two populations of OVA specific CD8^+^ T cells: endogenous dextramer dsRed^−^ and transferred dextramer dsRed^+^ cells (Fig. 7a). Both populations are severely reduced in the absence of SPARC in both frequencies (Fig. 7a) and number (Fig. 7b,c) despite similar distribution in the periphery (Fig. S5). Multiphoton imaging of dsRed^+^ T cells in the brain reveals dsRed^+^ cells in association with SHG signal in both the presence and absence of SPARC (Fig. 7d). However, there were fewer average cells migrating in the absence of SPARC (Fig. 7d), these cells did not travel as far (Fig. 7e, f), were significantly slower (Fig. 7g), and migrated shorter distances (Fig. 7h) than cells in a SPARC sufficient environment. Together, these data indicate that SPARC-dependent tissue remodeling during chronic inflammation in the CNS is required for efficient CD8^+^ T cell accumulation in the brain and early control of parasite burden.

## Discussion

Chronic *T. gondii* infection represents a somewhat harmonious balance between parasite and host with limited clinical pathology from either the pathogen or the immune response. This delicate balance is maintained through the controlled activation and migration of immune cells from the bloodstream to sites of infection within the CNS. Studies using multiphoton microscopy and second harmonic generation (SHG) have expanded upon the important role for ECM in facilitating cell migration into and through tissues^8,10,11,37^. This includes the discovery of a notable restructuring of tissue during *T. gondii* infection with the development of an infection-induced network of fibers revealed by SHG^37^. Although T cells were observed migrating along network strands, the composition, regulation, and the purpose of this change in tissue architecture is unknown. Here we demonstrate that ECM remodeling in the brain occurs early following infection, that it is dependent on the secreted matrix associated protein SPARC, and this interaction helps facilitate antigen-specific T cell migration in the brain parenchyma.

A time course and volumetric analysis reveals a maximum second harmonic signal at 2 weeks post infection (Fig. 3). This window, although occurring over the course of a few weeks, suggests induction is due to an initial infection-induced event rather than caused by long-term continuous inflammation in the brain. Invasion of parasites and the initial influx of immune cells occurs over this period and thus the development of this network may be due to changes in blood brain barrier. A considerable amount is happening at this site during this period. As *Toxoplasma* disseminates into the brain it can invade and replicate within endothelial cells^46^ and thus has the ability to cause direct damage to barrier integrity in addition to inflammatory-induced changes including upregulation of adhesion molecules and diapedesis of immune cells. Thus, our observation of parenchymal SHG emanating from blood vessels (Fig. 1b) may support the concept of an unraveling of ECM at the barrier which the production of SPARC helps to organize into structures to aid protective immune responses. As such structures are visible during other forms of neuroinflammation such as TBI in this instance, appropriate imaging may allow clinical observation of weakened vasculature or inflamed areas of the brain.

The ECM acts as a malleable substrate providing bioavailable chemokines, arrangement of cellular interactions, and a path from point A to point B. Its remodeling involves the deposition, enzymatic degradation and uptake of matrix proteins by cells such as fibroblasts and macrophages, and in the brain by astrocytes and microglia^67,68^. Therefore, the biological significance of SPARC’s interaction with chemotactic cytokines in the inflamed brain is probably multifaceted. First, binding to SPARC may help keep chemokines in a confirmation that will most effectively interact with their respective receptors on the surface of a migrating cell, eliciting optimal movement so that the cell can find its target efficiently. SPARC’s interaction with CCR7 ligands specifically may contribute to the maintenance of an extracellular gradient of either CCL21 or CCL19. Although CCL21 and CCL19 are both agonists of CCR7, only CCL19 is involved in receptor internalization through β-arrestin signaling^69,70^. Preference of SPARC for CCL19 may suggest a mechanism to avoid receptor desensitization away from sites of infection and to facilitate some degree of desensitization near sites of infection^70,71^. The presentation of CCR7 chemokines in this manner may facilitate T cells and dendritic cells to follow similar migratory routes into the tissue analogous to stromal cells in the lymph node^72^. While CXCL10 needs to be present for ideal migratory speed^34^, it does not necessarily need to exist in a gradient to exert its effects on T cells. At the highest CXCL10 concentrations, there may be some indication of a bi-phasic dose response that occurs between CXCL10 and SPARC. The fact that CXCL10 exists in a monomer–dimer equilibrium with a dissociation constant of approximately 9 μM may explain this observation although we cannot reach high enough concentrations of CXCL10 to test this hypothesis^59^. The two dimeric forms and one tetrameric form of CXCL10 in solution further complicates the binding assay. Moreover, the magnitude of the change in normalized fluorescence between the bound and unbound states is not far outside the noise of an individual measurement for interactions between CXCL10 and SPARC, so there is far less confidence in the calculated dissociation constant. This noise may arise from the multiple oligomeric forms of CXCL10 that exist; however, the results seem to indicate a minimal degree of interaction between CXCL10 and SPARC or binding that may be specific to one structural form of CXCL10.

In addition, SPARC may be needed to clear excess chemokine from the ECM regardless of the presence of a gradient, employing a Stabilin-1-mediated mechanism similar to the way SPARC facilitates collagen remodeling^48,73^. Alternatively, SPARC’s role in ECM remodeling likely helps to accommodate immune cell infiltration into the brain independently of its interaction with chemokines. Indeed, SPARC’s presence in our in vitro assays lead to chemokine-independent increased cell migration, which could support either scenario. Additional studies are necessary to determine the site(s) to which CCL21 and CCL19 bind to SPARC and whether those interactions would help or hinder SPARC’s other known functions.

In this study, we identify a novel role for SPARC in providing protective immunity during chronic *T. gondii* infection through regulation of infection-induced reticular fibers in the CNS. SPARC is highly expressed in all types of tissue during development and then limited to tissues with high ECM turnover after maturation^74^. In the adult brain, SPARC expression is normally limited to the cerebellum and brainstem and is thought to play a role in synaptic plasticity and neurogenesis^75^. Following infection with *T. gondii,* we observed an upregulation of SPARC in the frontal cortex of the brain, an area associated with parasite infection (Fig. 2)^76,77^. The peak of SPARC induction is prior to the major influx of immune cells that is observed around day 21. Upregulation of matrix metalloproteinases (MMP), key mediators of tissue remodeling, have been observed in the brain as early as 3 days following infection, suggesting that matrix remodeling in the CNS occurs during acute infection^78^. This early requirement is also seen in the decreased ability of SPARC−/− mice to control parasite burden with as much as a threefold increase in parasite DNA in the brain of SPARC−/− mice (Fig. 3d). This may be due to an increase in the ability of parasites to migrate from the vasculature into the brain^46^ in the absence of SPARC and perhaps the constitutive expression of SPARC in the cerebellum leads to a decrease in infection in this area^77^. However, our data also support reduced anti-parasitic T cell responses in the brain in the absence of SPARC. This is in contrast to mycobacterium infection where, despite SPARC’s critical role in granuloma formation, SPARC deficiency leads to enhanced immune responses^79^. Adoptive transfer of pre-activated parasite-specific CD8^+^ T cells demonstrates that the reduced accumulation of antigen-specific cells in the brain is not due to a failure of priming in the periphery (Fig. S5) but instead supports the model where SPARC is required for trafficking of T cells into or retention within the brain parenchyma and SPARC’s absence leads to a temporary loss of control of parasite burden. It is also possible that in the absence of SPARC and appropriate tissue remodeling there may be an increase in the number of bystander T cells able to migrate across the blood brain barrier. Thus, we propose that the function of SPARC in the brain following infection is to regulate the assembly and maintenance of the network of fibers, which in turn is required for early immune cell access and parasite control as chronic CNS infection is established.

The data point to a model where initial parasite invasion or detection of systemic inflammation leads to an upregulation of SPARC in the brain facilitating tissue remodeling around the vasculature and into the parenchyma. This, in cooperation with chemokines expressed by astrocytes in the parenchyma^36^, leads to efficient immune cell access to the CNS and parasite control. Once the parasite is encysted, the signals driving SPARC are lost and maintenance of immune cells and chronic infection no longer requires further tissue remodeling.

In summary, we have identified a novel role for the matricellular protein SPARC in the regulation of an infection-induced fibrous network in the CNS. SPARC, together with CCR7 ligands, is required for antigen-specific T cell accumulation and migration in the brain parenchyma to control parasite burden during early chronic infection.

## Supplementary Information

Supplementary Figures.

## Data Availability

The data generated during the current study are available from the corresponding author upon reasonable request.
