# Supplementary material for: SPARC coordinates extracellular matrix remodeling and efficient recruitment to and migration of antigen-specific T cells in the brain following infection
Source: Sci Rep. 2021 Feb 25;11:4549. doi: 10.1038/s41598-021-83952-0 (PMC7907143; doi:10.1038/s41598-021-83952-0)
Supplement: Supplementary file 1 — Supplementary Figures. [file 41598_2021_83952_MOESM1_ESM.pdf]

Supplementary Figures

SPARC coordinates extracellular matrix remodeling and efficient recruitment to and migration of antigen-specific T cells in the brain following infection.

Kathryn E. McGovern[1], J. Philip Nance[1], Clément N. David[1], Reed E. S. Harrison[2,] Shahani Noor[1], Danielle Worth[1], Tyler A. Landrith[1], Andre Obenaus[3] Monica J. Carson[1], Dimitrios Morikis[2], and Emma H. Wilson[1]#.

1. School of Medicine, Division of Biomedical Sciences, University of California, Riverside, CA

2. Department of Bioengineering University of California, Riverside, CA 92521-0129, USA

3. School of Medicine, University of California, Irvine, CA 92697, USA

#Address correspondence to Emma H. Wilson, emmaw@ucr.edu

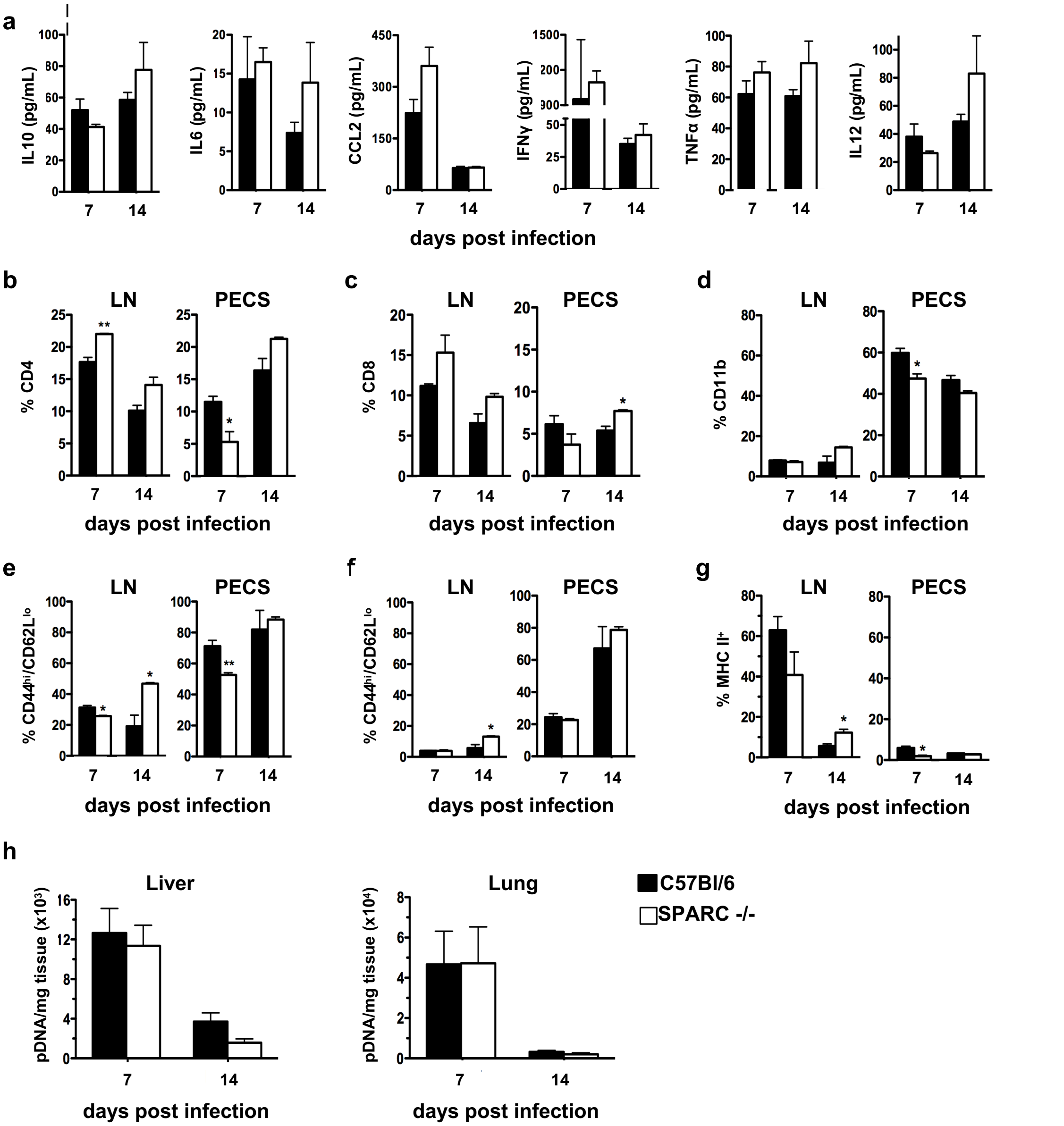

Supplemental Figure 1. SPARC-null mice have no defect during acute *T. gondii* infection. C57Bl/6 and SPARC -/- mice were infected with the Prugniaud strain of *T. gondii* and sacrificed at 7 and 14 days following infection. (a) Serum was isolated from whole blood samples and analyzed for IL-10, IL-6, MCP-1, IFNγ, TNFα, and IL-12 using a cytometric bead assay (CBA). (b-g) Cervical lymph nodes (LN) and peritoneal exudate cells (PECS) were harvested and prepared for analysis by flow cytometry. Cell suspensions were stained and analyzed for expression of (b) CD4^{+} T cells, (c) CD8 T cells, (d) monocytes (CD45^{hi}/CD11b^{+}), (e) activation of CD4^{+} T cells (CD44^{hi}/CD62^{lo}), (f) activation of CD8 T cells (CD44^{hi}/CD62^{lo}), and (g) MHC II expression by CD11b^{+} cells. (h) DNA was isolated from the liver and lung and analyzed for parasite burden using qPCR. Results are displayed as parasites per mg tissue. Data are representative of at least 2 individual experiments with a minimum of n=4 and are displayed as mean ± SEM, * $p < 0.05$, ** $p < 0.01$.

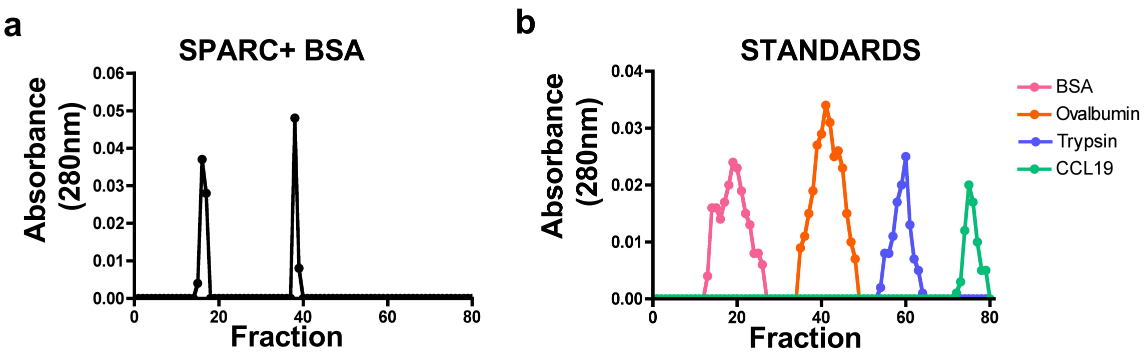

Supplemental Figure 2. Controls for measuring SPARC's interaction with CCR7 ligands. (a) Recombinant SPARC was incubated with BSA and the mixture and applied to a Sephadex G-50 column. The elution profiles show two peaks in the expected fractions based on each protein's molecular weight, indicating no interaction. (b) Standard proteins were run through the column to determine where proteins or protein complexes should elute based on molecular weight.

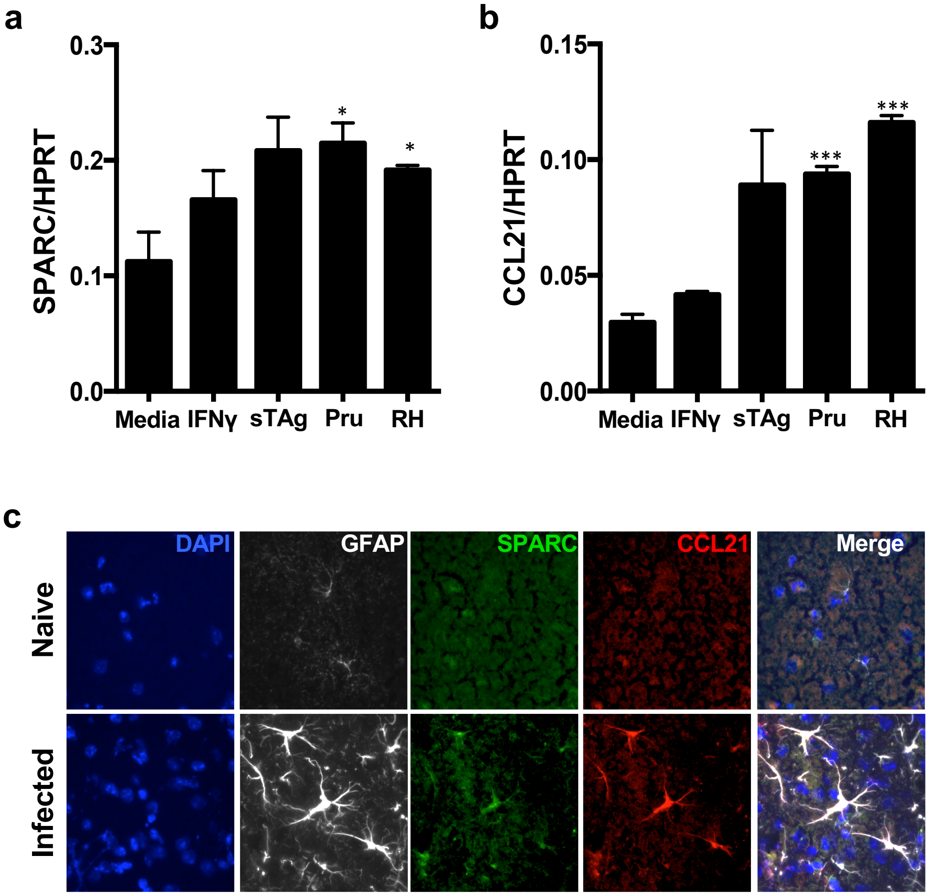

Supplemental Figure 3. SPARC and CCL21 are expressed by murine astrocytes upon infection. (a and b) Primary astrocyte cultures were infected with the Pru or RH strain of *T. gondii*, or stimulated with sTAg, the cytokine IFNγ, or media alone. RT-qPCR was conducted to determine the absolute copy number of CCL21 and SPARC transcripts compared to those of the reference gene HPRT using a standard curve. (c) Brains of naïve and chronically infected mice were stained for GFAP (white), SPARC (green), and CCL21 (red).

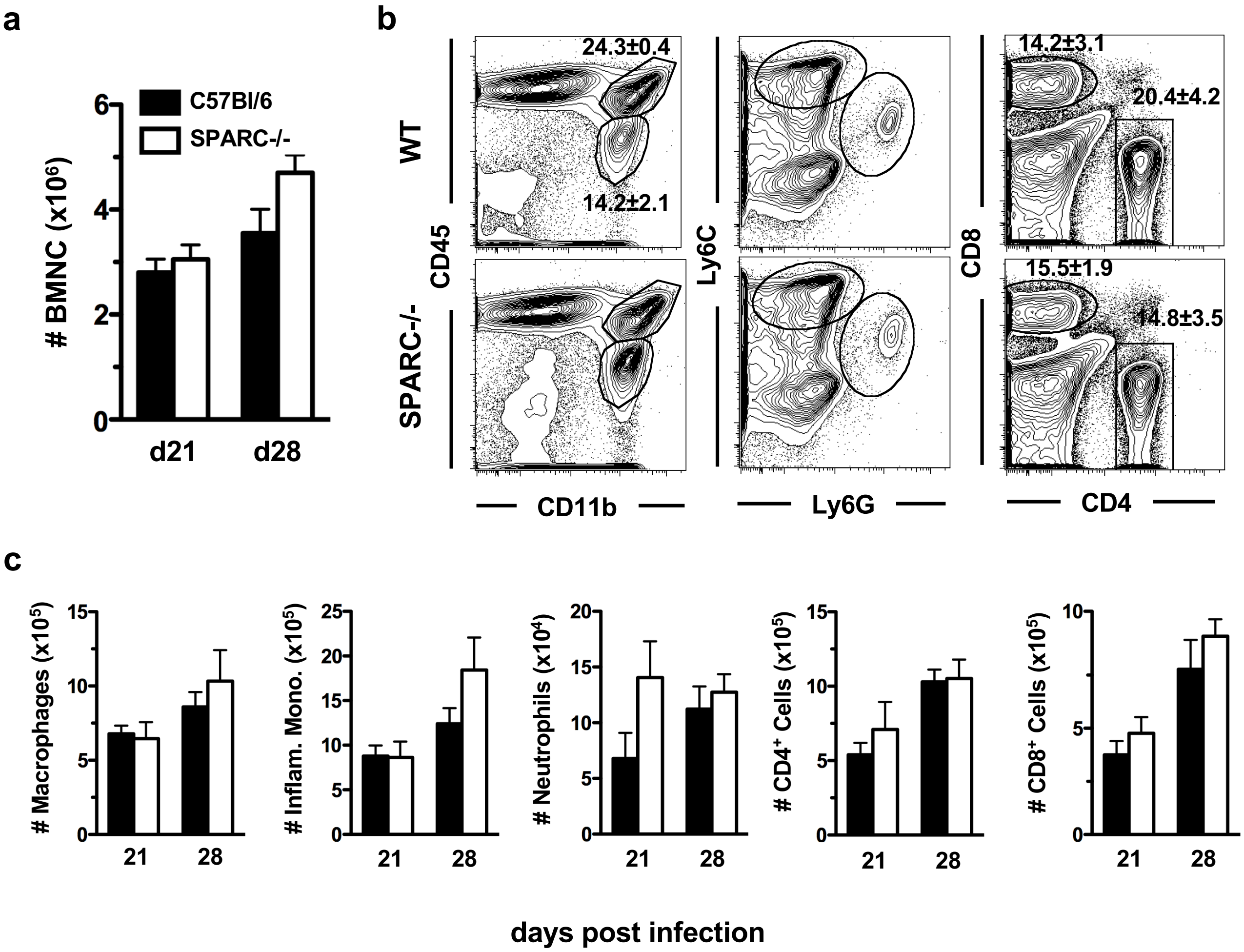

Supplemental Figure 4. SPARC-dependent and -independent immune cell migration to the brain during chronic infection. C57Bl/6 and SPARC -/- mice were infected with Pru-OVA and sacrificed at 21 and 28 days following infection. Brains were harvested and BMNCs analyzed by flow cytometry. (a-c) for CD4$^+$ T cells, CD8 T cells, macrophages (CD45^{hi}/CD11b$^+$), microglia (CD45^{int}/CD11b$^+$), inflammatory monocytes (IM) (Ly6G^{lo}/Ly6C^{hi}) and neutrophils (Ly6G^{hi}/Ly6C^{int}). (a) Total number of BMNCs isolated from the brain. (b) Percentages of (left) macrophages and microglia, (center) IMs and neutrophils, and (right) CD4 and CD8 T cells in C57Bl/6 and SPARC-/- at 21 days p.i. (c) Absolute cell counts of macrophages, IMs, neutrophils, and CD4$^+$ and CD8$^+$ T cells, at days 21 and 28 p.i. (d) Proportion and number of OVA-specific CD8$^+$ T cells recovered from the brain at 28 days post infection. Data are representative of at least 2 individual experiments with a minimum of n=4, were analyzed using the student's T test and are represented as mean $\pm$ SEM.

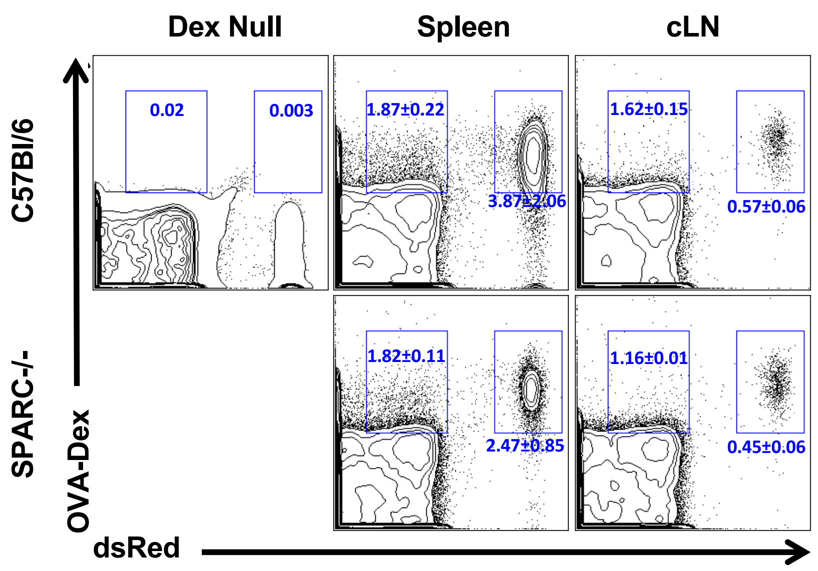

Supplemental Figure 5. Reduced antigen-specific T cell recruitment to the brain is independent of priming in the periphery.
C57Bl/6 and SPARC -/- mice were infected with OVA expressing parasites. 21 days following infection mice received $5x10^6$ million activated OTI-dsRed$^+$CD8$^+$ T cells i.v. Spleens and cervical lymph nodes (cLN) were harvested one week later and single cell suspensions were prepared for analysis by flow cytometry. Proportions of endogenous OVA-specific CD8$^+$ T cells and transferred dsRed$^+$ OVA-specific CD8$^+$ T cells are shown. Data are representative of at least 2 individual experiments with a minimum of n=3, were analyzed using the student's T-test and are represented as mean ± SEM.

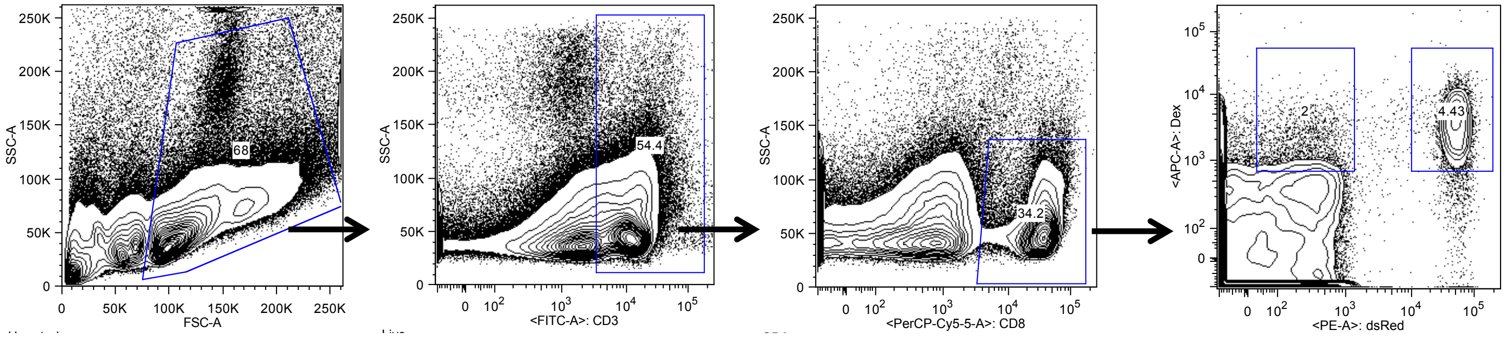

Supplemental Figure 6. Gating scheme for flow cytometry data featured in figure 7 and supplemental figure 6. Leukocytes from brain, spleen, and cervical lymph nodes were isolated and incubated with antibodies to CD3-FITC, CD8-PerCP-Cy5.5, and OVA-dextramer-APC to determine the population of transferred (dsRed⁺) OVA specific T cells present in each organ.

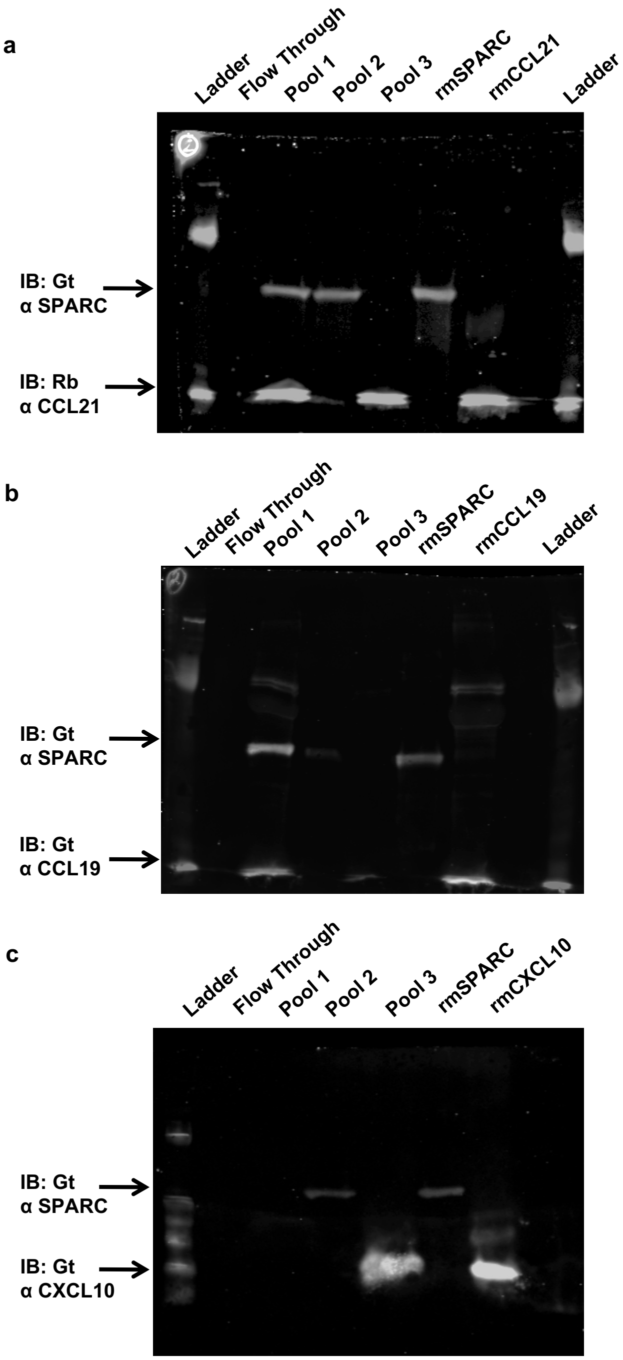

Supplemental Figure 7. Uncropped western blots featured in figure 4. To determine if SPARC interacts with the major chemokines that dictate cell trafficking in the brain, recombinant murine SPARC, CCL21, CCL19, CXCL10 (5ug each, R&D Systems or Peprotech) or BSA (Sigma, St. Louis, MO) were resuspended in 70 μL phosphate buffered saline (PBS) with 2mM Calcium, mixed in the pairs indicated in figure 4, and filtered using Sephadex G50 at 4°C. Absorbance of eluted fractions at 280nm were recorded and fractions that contained protein were pooled and concentrated with Amicon filters (Millipore, Billerica, MA). Concentrated eluate was mixed with Laemmli buffer and subjected to SDS-PAGE on 10% Tris-HCL gels (Biorad, Hercules, CA) followed by western blot onto nitrocellulose membranes on a semi-dry blotter (Biorad, Hercules, CA). Membranes were blocked with 5% nonfat dry milk then probed with anti-SPARC (R&D Systems), anti-CCL21 (Peprotech), anti-CCL19 (R&D Systems) or anti-CXCL10 (R&D Systems) antibodies. Blots were imaged using an Odyssey Infrared Imaging system (Li-COR Biosciences).
